# Supplementary material for: Preterm nutritional intake and MRI phenotype at term age: a prospective observational study
Source: BMJ Open. 2014 May 23;4(5):e005390. doi: 10.1136/bmjopen-2014-005390 (PMC4039783; doi:10.1136/bmjopen-2014-005390)
Supplement: Author's manuscript [file bmjopen-2014-005390.draft_revisions.pdf]

BMJ Open

# The influence of preterm nutrition upon magnetic resonance imaging phenotype at term age: A prospective observational study.

| | |
|---|---|
| Journal: | *BMJ Open* |
| Manuscript ID: | bmjopen-2014-005390 |
| Article Type: | Research |
| Date Submitted by the Author: | 02-Apr-2014 |
| Complete List of Authors: | Vasu, Vimal; William Harvey Hospital, Child Health<br>Durighel, Giuliana; Imperial College, Institute of Clinical Sciences<br>Thomas, E Louise; Imperial College, Metabolic and Molecular Imaging Group<br>Malamateniou, Christina; Kings College, Division of Imaging Sciences<br>Bell, Jimmy; Imperial College London, Metabolic and Molecular Imaging Group<br>Rutherford, Mary; Kings College, Division of Imaging Sciences; Imperial College, MRC Clinical Sciences Centre<br>Modi, Neena; Imperial College London, Neonatal Medicine |
| <b>Primary Subject Heading</b>: | Paediatrics |
| Secondary Subject Heading: | Nutrition and metabolism, Paediatrics, Radiology and imaging |
| Keywords: | NEONATOLOGY, Neonatal intensive & critical care < INTENSIVE & CRITICAL CARE, Magnetic resonance imaging < RADIOLOGY & IMAGING |
| | |

SCHOLARONE™
Manuscripts

For peer review only

**Title:**The influence of preterm nutrition upon magnetic resonance imaging phenotype at term age: A prospective observational study.

**Authors:** Vimal Vasu[1,5] MBBS MD, Giuliana Durighel[2] MSc, Louise Thomas[2] PhD, Christina Malamateniou[3,4] PhD, Jimmy D Bell[2] PhD, Mary A Rutherford[3,4] MBBS MD, Neena Modi[1] MBChB MD

**Affiliations:** [1]Section of Neonatal Medicine, Department of Medicine, Imperial College London, Chelsea & Westminster Hospital campus, 369 Fulham Road, London, SW10 9NH, UK; [2]Metabolic and Molecular Imaging Group, MRC Clinical Sciences Centre, Imperial College London, London, W12 0HS, UK; [3]Division of Clinical Sciences, Imperial College London, MRC Clinical Sciences Centre Hammersmith Hospital, Du Cane Road, London, United Kingdom, W12 0HS, UK; [4] Centre for the Developing Brain, Department of Perinatal Imaging & Health, Division of Imaging Sciences & Biomedical Engineering, Faculty of Medicine, King's College London, St Thomas' Hospital, London SE1 7EH, UK; [5] Department of Child Health, William Harvey Hospital, Ashford, Kent, TN24 0LZ, UK.

**Address Correspondence to:** Dr Vimal Vasu, Department of Child Health, William Harvey Hospital, Ashford, Kent, TN24 0LZ, United Kingdom. Email: vimal.vasu@nhs.net

**Short Title:** Preterm nutrition and phenotype

For peer review only

**Abbreviations:** ACA-anterior cerebral artery, AT-adipose tissue, CAVT-cerebral arterial vessel tortuosity, CRIB II-Clinical Risk Index for Babies II, CSF-Cerebrospinal fluid, DSC-deep subcutaneous, I-internal, IHCL-intrahepatocellular lipid, MCA-middle cerebral artery, MR-magnetic resonance, NMR-nuclear magnetic resonance, PMA-post menstrual age, PCA-posterior cerebral artery, SDS- standard deviation scores, SSC-superficial subcutaneous

**Key Words:** preterm, nutrition, body composition, magnetic resonance

For peer review only

## ABSTRACT

**Objective** To describe (i) the relationship between nutrition and the preterm-at-term infant phenotype, (ii) phenotypic differences between preterm-at-term infants and healthy term born infants and (iii) relationships between somatic and brain MRI outcomes.

**Design** Prospective observational study.

**Setting** UK tertiary neonatal unit.

**Participants** Preterm infants (<32 weeks gestation) (n=22) and healthy term infants (n=39)

**Main outcome measures** Preterm nutrient intake; total and regional adipose tissue (AT) depot volumes; brain volume and proximal cerebral arterial vessel tortuosity (CAVT) in preterm infants and in term infants.

**Results** Preterm nutrition was deficient in protein, high in carbohydrate and high in fat. Preterm nutritionwas not related to AT volumes, brain volume or proximal CAVT score; a positive association was noted between human milk intake and proximal CAVT score (r=0.44, *p*=0.05). In comparison to term infants, preterm infants had increased total adiposity, comparable brain volumes and reduced proximal CAVT scores. There was a significant negative correlation between deep subcutaneous abdominal adipose tissue volume and brain volume in preterm infants (r= -0.58, *p*=0.01).

**Conclusions** Though there are significant phenotypic differences between preterm infants at term and term infants, preterm macronutrient intake does not appear to be a determinant. Human milk may have beneficial effects upon cerebrovascular


For peer review only

development. The negative correlation between adiposity and brain volume mirrors findings in adults and may suggest that adiposity induced inflammation maybe one mechanism contributing to neurocognitive impairment.

For peer review only

## ARTICLE SUMMARY

Provision of nutrition to preterm infants is complicated by immaturity, critical illness, and a limited evidence base relating early nutrition to functional health outcomes.

- In comparison to term infants, preterm infant phenotype at term is characterized by adiposity, comparable brain volume, increased CSF volume and reduced cerebral artery tortuosity.

- Though preterm macronutrient intake is not a determinant of this phenotype, human milk intake is positively associated with cerebral artery tortuosity.

- In preterm infants, regional adiposity is negatively correlated with brain volume measured at term age.

### Strengths of study

- No previously published studies have studied the relationship between preterm nutritional intake and MRI outcomes at term age.

- omprehensive ascertainment of preterm nutritional data in parallel with somatic and brain MRI.

- Use of term born infants as comparator for MRI outcomes.

For peer review only

**Limitations of study**

- Limited sample size.
- Prospective observational nature of study.

For peer review only

## INTRODUCTION

Preterm nutritional guidelines are based upon consensus expert opinion (1,2) rather than compelling evidence and the “optimal” diet for long term health remains unknown (3). Though there is now greater clinical emphasis upon preterm nutrition, protein deficiency remains a significant risk and our previously published data in 18 preterm infants from a single centre indicate that the preterm diet is low in protein whilst being high in both carbohydrate and fat (4). Our data also suggest that preterm macronutrition may affect later health by demonstrating a positive association between first week lipid intake in preterm infants and elevated intrahepatocellular lipid (IHCL), which in adults is associated with the cardio-metabolic syndrome (5).

The preterm phenotype at term is characterized by aberrant adipose tissue partitioning (6), reduced proximal cerebral arterial vessel tortuosity (CAVT) (7), reduced deep grey matter volumes (8), and reduced cerebral cortical folding (9). The somatic phenotype observed is of concern as adiposity is associated with inflammation and reduced brain volume in the adult population (10).

Here we present prospective observational data designed to (i) assess the influence of nutrition upon the preterm phenotype at term age (ii) describe phenotypic differences between preterm infants at term and term healthy infants and (iii) examine relationships between somatic and brain MRI measurements. The a priori hypotheses of our study were that (i) preterm macronutrient intake would be positively associated with central nervous system phenotype (brain volume & cerebral vessel tortuosity)

For peer review only

and (ii) preterm macronutrient intake would be negatively associated with internal abdominal (visceral) adiposity. The relationship between somatic and brain MRI outcomes represents a post hoc exploratory analysis.

For peer review only

## PATIENTS AND METHODS

Following research ethics approval (REC 07/Q0403/46) and informed parent consent, preterm infants admitted to Chelsea and Westminster Hospital neonatal unit, London, UK (< 32 weeks of gestation) and term infants (37-42 weeks gestation) on the postnatal ward were recruited (January 2007-July 2008). Infants with congenital anomalies were excluded.

### Preterm nutrition and growth

Preterm nutritional practice during the study period has been previously described (4). In brief, enteral feeds were commenced on day 1 with either maternal expressed breast milk or donor expressed breast milk prior to consideration of the use of formula. Human milk fed infants received Nutriprem breast milk fortifier (Cow & Gate) once 150ml/kg/day of feed volume was reached. Parenteral nutrition was commenced on the day of birth.

Weight and head circumference data for the preterm group are expressed as SDS at birth and at time of MRI. Birth length was not routinely measured during the study period. Macronutrient and human milk intake was recorded between birth and $34^{+6}$ weeks PMA using a nutritional data capture system designed in-house (Nutcracker™, Imperial College, London, UK). Macronutrient data were expressed as the difference between recommended daily intake (RDI) from Tsang et al (i) and actual daily intake in g or kcal/kg for the period of either early (first week of life) or total nutrition (birth and $34^{+6}$ PMA) (iv). Human milk intake is expressed as ml/kg/day and as percentage of enteral feeds given as human milk.

For peer review only - http://bmjopen.bmj.com/site/about/guidelines.xhtml

**Magnetic Resonance Imaging**

MRI was performed at the Robert Steiner Unit, Hammersmith Hospital shortly after discharge from the neonatal unit as previously described (iv) using a Philips Achieva system (Best, the Netherlands).

*(i) Total and Regional AT*

Imaging parameters are shown in Table 1. Regional AT depots were classified as total superficial subcutaneous, total deep subcutaneous and total internal and subdivided into abdominal or non-abdominal (Figures 1 & 2). AT volumes were calculated as previously described (6). Non AT mass was calculated by conversion of AT volume to AT mass (density of AT of 0.9 grams/cm$^3$) and subtraction of the result from weight at time of MRI.

*(ii) Brain volume*

T2 weighted images were acquired using a dynamic sequence of six separate loops of single shot images which were then registered and reconstructed to produce volumetric datasets to eliminate the effects of motion artefact (11). Imaging parameters are shown in Table 1. Images were corrected for Radiofrequency inhomogeneity (http://mipav.cit.nih.gov). BET Brain extraction tool FSL Version 3 (http://www.fmrib.ox.ac.uk/fsl/) (12) was then used to delete non brain tissue and create binary brain masks representing intracranial volume (13). A mask of ventricular and CSF spaces was created using the thresholding feature of Image J, a java based image processing program (14). Brain volume was calculated by subtraction of the volume of the ventricular and CSF mask from the volume of the

For peer review only

intracranial mask using ImageJ (Figure 3). Brain MRIs were reported by MR for clinical purposes and given a score (0-13) adapted from Dyet et al (15) so that if necessary, pathological findings could be accounted for.

*(iii) Proximal cerebral arterial vessel tortuosity (CAVT) measurement and analysis*

An optimized neonatal three dimensional time of flight MRA sequence was used to assess the anterior, middle and posterior cerebral arteries (7). Imaging parameters are shown in Table 1. Vessel tortuosity was assessed using a previously validated measurement, distance factor (16) (Figure 4) and a CAVT score was determined as a global measure of tortuosity for each subject by calculating the mean of the ACA, MCA and PCA distance factor.

**Illness severity**

CRIB II Score was calculated in preterm infants (17).

**Sample size calculation and analyses**

Based on previous work (6), we estimated that recruitment of 60 PT and 60 T infants allowed detection of a 0.5 SD difference between the groups for AT volume (Power 80%, Significance 5%). As this was an exploratory hypothesis-generating prospective observational study and given the uncertainty as to what differences were of clinical importance, additional sample size calculations were not considered. Data were adjusted for relevant confounding variables where appropriate and are presented for each of the MR outcomes with a comparison of outcomes between the groups. Data were analyzed by comparison of means and tested for normality. Parametric or non parametric methods were then applied accordingly. Within the preterm group, Pearson

For peer review only

Correlation was used to investigate the relationship between nutrition and MR outcomes at term age and these were limited to the patients in who both nutritional data and the MRI outcome were successfully acquired. For a multivariate analysis a minimum of 10n subjects is considered appropriate, where n is the number of covariates. Data are presented as mean (SD) or mean (95% CI).

For peer review only

## RESULTS

61 infants were recruited during the study (Preterm 22, Term 39). MR images without motion artefact were acquired as follows: AT volume (preterm 22, term 39), brain volume (preterm 19, term 19), CAVT measurement (preterm 20, term 13). In preterm infants, mean (SD) CRIB II score was 7.6 (4.2). 25% had chronic lung disease of prematurity (defined as an oxygen requirement at 36 weeks PMA), 20% had a patent ductus arteriosus requiring pharmacological therapy, 5% had retinopathy of prematurity requiring laser therapy and 5% had a large intraventricular haemorrhage (Grade III/IV Papile classification). 0% had necrotising enterocolitis requiring surgery. Table 2 summarises the baseline and imaging characteristics and the MR outcomes of the preterm and term groups.

### Preterm nutrition & growth

Growth and nutrition data for the preterm cohort have been previously published (4). In brief, mean (SD) for birth weight and birth head circumference SDS were: -0.13 (0.78) and -0.64 (1.12). At time of MRI, the respective values were: -1.39 (0.93) and -0.86 (1.33). Weight gain was mean (SD) 9.48 (1.73) g/kg/day between birth and $34^{+6}$ weeks. Preterm macronutrient intakes for both the first week after birth and for the period from birth until $34^{+6}$ weeks post menstrual age revealed a mean protein deficit (first week: -1.6g/kg/day; birth until $34^{+6}$ weeks -0.4g/kg/day) in the context of excessive carbohydrate and fat intake. Mean (SD) human milk intakes for these periods were 36.6 (29.7) ml/kg/day and 108.9 (46.6) ml/kg/day respectively.

For peer review only

**Preterm Nutrition and MRI Outcomes**

*(i) Human milk and MRI outcomes*

After adjustment for weight at MRI, there was no correlation between early or total human milk intake and either regional AT volumes or brain volume at term age. There was no correlation between early human milk intake and overall CAVT score (r= 0.31, p=0.18); however, there was a weak positive relationship between total human milk intake and overall CAVT score (r=0.44, p=0.05) (Figure 5). This relationship was also apparent when total human milk intake was expressed as percentage of total milk intake (r=0.45, p=0.04).

*(ii) Macronutrient intake and MRI outcomes*

There were no correlations between macronutrient intake (protein, carbohydrate, fat) and total adiposity (n=22). After adjustment for PMA, neither early nor total preterm macronutrient intake were correlated with brain volume at term age (n=19). There were no relationships noted between early or total macronutrient intake and proximal CAVT score (n=20), a summary measure of tortuosity.

**Preterm at Term versus Term Group Comparison**

*(i) Anthropometry*

Anthropometric data were adjusted for PMA at time of MRI. Preterm infants were smaller, shorter and had smaller head circumferences than term infants (Table 2).

For peer review only

*(ii) Adiposity*

Adipose tissue data were adjusted for PMA at time of MRI. Preterm infants had more AT than term infants with expansion of the superficial subcutaneous, deep subcutaneous and internal AT depots. There was a parallel reduction in non adipose tissue mass (Table 2).

*(iii) Brain volume*

Brain MRI scores in the cohort of preterm infants were mean (SD) 3.6 (2.1). There was no correlation between brain MRI score and brain volume (r=0.31, $p$=0.20). At term age, preterm brain volumes were smaller than term infants [PT 461.74 (436.16-487.32) ml v 493.74 (476.43-511.05) ml, $p$=0.05. However, after adjustment for weight at time of MRI, a significant confounding factor, there was no difference between the groups. Preterm-at-term infants had significantly increased CSF volume (Table 2).

*(iv) Distance Factor & Proximal CAVT score*

In comparison to term born infants, preterm-at-term infants had a significant reduction in cerebral arterial vessel tortuosity (Table 2).

**Relationships between somatic and central nervous system MRI phenotype**

After adjustment for weight at MRI, there was a significant negative correlation noted in preterm at term infants but not in term infants, between deep subcutaneous AT volume and brain volume (r=-0.58, $p$=0.01) (Table 3). There were no statistically significant correlations between regional AT and CAVT score.

For peer review only

**DISCUSSION**

We identify novel findings of interest including a positive association between human milk intake and proximal CAVT, a marker of cerebrovascular development and a negative correlation between regional adipose tissue volume and brain volume in preterm infants. The study also comprehensively characterises the somatic and brain phenotype of a cohort of preterm infants at term in comparison to term born healthy infants and demonstrates (i) reduced anthropometric measures (ii) increased total and regional adiposity (iii) reduced non-adipose tissue mass (iv) comparable brain volumes (v) increased CSF volume and (vi) reduced proximal cerebral arterial vessel tortuosity. We have shown no relationship with either body composition or brain volume at term age within the range of macronutritional intakes received by the preterm infants in this study.

Key study strengths include the comprehensive ascertainment of preterm nutritional data and the assessment of a number of MR outcomes. Limitations include the prospective observational design of the study that preclude any inferences regarding causality, and the “sub-optimal” preterm nutritional intake, a potential determinant of the observed phenotype. Successful acquisition of a number of different MR outcomes without use of sedation was challenging and motion artefact meant that not all recruited infants had data of sufficient quality for analysis. We were unable to recruit the desired number of infants and recognise that the study may be underpowered. The preterm infants studied were relatively healthy, as evidenced by low illness severity (CRIB II scores), low incidences of serious neonatal morbidity

For peer review only

and low brain MR scores, factors that may have attenuated any associations with nutritional intake.

The finding of increased adiposity in preterm infants confirms our previous work (6). We have previously shown expansion of the internal abdominal AT compartment in preterm at term infants in comparison to term born infants in a cohort of preterm infants recruited in 2002-2003 (6), a time at which neonatal nutrition was possibly not as carefully considered as it is today. This contrasts with our present data which demonstrates a global expansion of all AT depots. Whether the differences observed in AT partitioning between these studies relate to changes in nutritional practises and the provision of a more calorie dense diet is a plausible but as yet unproven hypothesis. Other potential mechanisms, which we have not explored, that might explain the increased adiposity seen in preterm infants include weight cycling and inflammation. It is known that cycling between high and low calorie diets (weight cycling) (18) results in the preferential deposition of AT over non AT mass (19) and this phenomenon often occurs in preterm infants when enteral feeds are discontinued because of concerns regarding feed intolerance and then restarted. Inflammation is also a known determinant of adiposity (20) and it is possible that the pro-inflammatory milieu often present in the perinatal period (maternal chorioamnionitis, use of intravenous lipid formulations high in omega-6 fatty acids and postnatal infection/ inflammation) may be relevant to the observed phenotype.

The nutritional intake received by this cohort of preterm infants was imbalanced (low in protein and high in both fat and carbohydrate) and sub-optimal in relation to expert consensus recommendations (1,2). In animal models, protein deficiency is associated

For peer review only

with a number of adverse health outcomes including a reduction in life span (21), cardiovascular dysfunction (22), reduced dendritic spine density (23), reduced brain weight (24) and reduced cortical blood vessel density (25). Human adult data indicate that dietary protein is an important factor in body weight regulation (26). Recent data in human ex-preterm infants suggests an association between early growth patterns and fractional anisotropy, a measure of brain microstructure (27). Our group have previously shown that preterm at term AT deposition can be attenuated by use of fortified human milk (28). Whether this translates into longer term benefit is unknown.

The negative correlation we show between adiposity in preterm at term infants and brain volume is consistent with findings in adults and children (10,29,30). This notwithstanding preterm at term brain volume was comparable to that of term born healthy infants which is also in keeping with previously published work (31). This, together with the finding of maintained head circumference SDS between birth and time of imaging may be indicative of “brain sparing” in nutritionally compromised infants.

We have confirmed the finding of reduced proximal CAVT in preterm infants (7). Though the natural history and long term neurodevelopmental sequelae of reduced CAVT are unknown, epidemiological data indicate that advancing gestation confers a significant reduction in risk of fatal adult cerebrovascular disease (occlusive stroke) (32). Our observation that human milk may be protective despite low macronutrient density, suggests that non-nutritive factors, such as vascular endothelial growth factor may play an important role in cerebrovascular development.

For peer review only

In conclusion, we have extended the characterization of the preterm-at-term phenotype. Our data do not support an association between macronutrient intake and body composition or brain volume. Other plausible determinants that remain to be explored are the roles of micronutrient deficiency, weight cycling, disease severity, and chronic inflammation.

For peer review only

**Funding Sources:** United Kingdom Medical Research Council; Chelsea and Westminster Hospital NHS Foundation Trust

**Financial Disclosure:** None

**Conflict of Interest:** None

**Clinical Trials Registration:** Not Applicable

**Data sharing statement:** No additional data available

**Contributorship Statement:** VV helped design the original prospective study, analysed the brain MRI data, drafted the initial manuscript and approved the final manuscript as submitted. GD optimized MR imaging parameters for the study. LT coordinated analysis of the adipose tissue MRI data. CM optimised MR angiography and advised on vessel tortuosity measurements. JB was involved in study design and supervised analysis of the adipose tissue MRI data. MR was involved in study design and supervised analysis of brain MRI data. NM was involved with study design and supervised the analysis of neonatal nutritional data. All authors critically reviewed and revised the manuscript and approved the final manuscript as submitted.

For peer review only

## REFERENCES


1. Tsang R, Uauy R, Koletzko B et al. Nutrition of the preterm infant: Scientific basis and practical guidelines. 2nd ed. Cincinnati, OH: Digital Education Publishing, Inc; 2005: 415-416.

2. Agostoni C, Buonocore G, Carnielli VP et al. Enteral nutrient supply for preterm infants: commentary from the European Society of Paediatric Gastroenterology, Hepatology and Nutrition Committee on Nutrition. *J Pediatr Gastroenterol Nutr*. 2010;50(1):85-91.

3. Vasu V, Modi N. Assessing the impact of preterm nutrition. *Early Hum Dev*. 2007;83(12):813-818.

4. Vasu V, Thomas EL, Durighel G et al. Early Nutritional Determinants of Intrahepatocellular Lipid Deposition in Preterm Infants at Term Age. *Int J Obes (Lond)*. 2013;37(4);500-504.

5. Mehta SR, Godsland IF, Thomas EL et al. Intrahepatic insulin exposure, intrahepatocellular lipid and regional body fat in nonalcoholic fatty liver disease. *J Clin Endocrinol Metab*. 2012;97(6):2151-2159.

6. Uthaya S, Thomas EL, Hamilton G et al. Altered adiposity after extremely preterm birth. *Pediatr Res*. 2005;57(2):221-225.


For peer review only

7. Malamateniou C, Counsell SJ, Allsop JM et al. The effect of preterm birth on neonatal cerebral vasculature studied with magnetic resonance angiography at 3 Tesla. *Neuroimage*. 2006;32(3):1050-1059.

8. Srinivasan L, Dutta R, Counsell SJ et al. Quantification of deep gray matter in preterm infants at term-equivalent age using manual volumetry of 3-tesla magnetic resonance images. *Pediatrics*. 2007;119:759-765.

9. Ajayi-Obe M, Saeed N, Cowan FM et al. Reduced development of cerebral cortex in extremely preterm infants. *Lancet*. 2000;356(9236):1162-1163.

10. Debette S, Wolf C, Lambert JC et al. Abdominal obesity and lower gray matter volume: a Mendelian randomization study. Neurobiology of Aging. 2014;35(2):378-386. Epub 2013 Aug 31.

11. Jiang S, Xue H, Glover A et al. MRI of moving subjects using multislice snapshot images with volume reconstruction (SVR): application to fetal, neonatal, and adult brain studies. *IEEE Trans Med Imaging*. 2007;26(7):967-980.

12. Smith SM, Jenkinson M, Woolrich MW et al. Advances in functional and structural MR image analysis and implementation as FSL. *Neuroimage*. 2004;23 Suppl 1:S208-219.

For peer review only

13. Smith SM. Fast robust automated brain extraction. *Hum Brain Mapp*. 2002;17(3):143-155.

14. Rasband, W.S. ImageJ, U. S. National Institutes of Health, Bethesda, Maryland, USA, http://imagej.nih.gov/ij/, 1997-2012.

15. Dyet LE, Kennea N, Counsell SJ et al. Natural History of Brain Lesions in Extremely Preterm Infants Studied With Serial Magnetic Resonance Imaging From Birth and Neurodevelopmental Assessment. *Pediatrics*. 2006;118:536 - 548.

16. Bullitt E, Gerig G, Pizer SM et al. Measuring tortuosity of the intracerebral vasculature from MRA images. *IEEE Trans Med Imaging*. 2003;22(9):1163-1171.

17. Parry G, Tucker J, Tarnow-Mordi W. CRIB II : an update of the Clinical Risk Index for Babies score. *Lancet*. 2003;361:1789-1791.

18. Brownell KD, Greenwood MR, Stellar E et al. The effects of repeated cycles of weight loss and regain in rats. *Physiol Behav*. 1986;38(4):459-464.

19. Rodin J, Radke-Sharpe N, Rebuffé-Scrive M et al. Weight cycling and fat distribution. *Int J Obes*. 1990;14:303-310.

20. So PW, Herlihy AH, Bell JD. Adiposity induced by adenovirus inoculation. *Int J Obes (Lond)*. 2005;29(6):603-606.

For peer review only

21. Ozanne SE, Hales CN. Lifespan: Catch-up growth and obesity in male mice. *Nature*. 2004;427(6973):411-412.

22. Cleal JK, Poore KR, Boullin JP et al. Mismatched pre- and postnatal nutrition leads to cardiovascular dysfunction and altered renal function in adulthood. *PNAS*. 2007;104(22):9529-9533.

23. Garcia-Ruiz M, az-Cintra S, Cintra L et al. Effect of protein malnutrition on CA3 hippocampal pyramidal cells in rats of three ages. *Brain Res*. 1993;625(2):203-12.

24. Perez-Torrero E, Torrero C, Salas M. Effects of perinatal undernourishment on neuronal development of the facial motor nucleus in the rat. *Brain Res*. 2001;905:54-62.

25. Bennis-Taleb N, Remacle C, Hoet JJ et al. A Low-Protein Diet During Gestation Affects Brain Development and Alters Permanently Cerebral Cortex Blood Vessels in Rat Offspring. *J Nutr*. 1999;129(8):1613-1619.

26. Westerterp-Plantenga MS, Lejeune MP, Nijs I et al. High protein intake sustains weight maintenance after body weight loss in humans. *Int J Obes Relat Metab Disord*. 2004:28(1):57–64.

27. Vinall J, Grunau RE, Brant R et al. Slower postnatal growth is associated with delayed cerebral cortical maturation in preterm newborns. *Sci Transl Med*. 2013;5(168):168ra8.

For peer review only

28. Uthaya S, Thomas EL, Bell J et al. Improved growth and adipose tissue partitioning in preterm infants receiving exclusive enteral feeds of fortified human milk in comparison with infants receiving human milk and formula.Proceedings of the Neonatal Society. 2007. (http://www.neonatalsociety.ac.uk/abstracts/uthayas_2007_growthfortifiedfeeds.shtml - 7KB)

29. Yau PL, Castro MG, Tagani A et al. Obesity and metabolic syndrome and functional and structural brain impairements in adolescence. Pediatrics. 2012;130(4):e856-864.

30. Debette S, Beiser A, Hoffmann U et al. Visceral fat is associated with lower brain volume in healthy middle-aged adults. *Ann Neurol*. 2010;68(2):136-144.

31. Boardman JP, Counsell SJ, Rueckert D et al. Early growth in brain volume is preserved in the majority of preterm infants. *Ann Neurol*. 2007;62(2):185-192

32. Koupil I, Leon DA, Lithell HO. Length of gestation is associated with mortality from cerebrovascular disease. *J Epidemiol Community Health*. 2005;59:473-474.

For peer review only

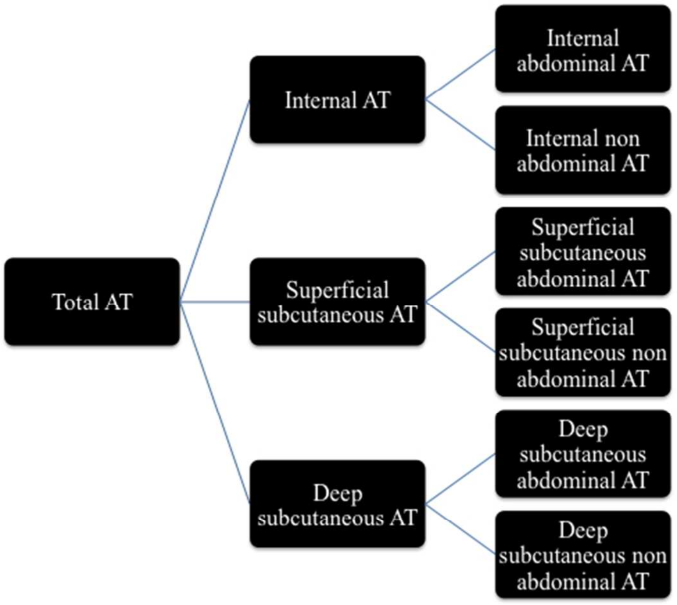


**Figure 1**. Classification of adipose tissue (AT) depots into internal, superficial subcutaneous and deep subcutaneous depots and further sub-classification according to abdominal or non abdominal position.

254x190mm (72 x 72 DPI)

iew only

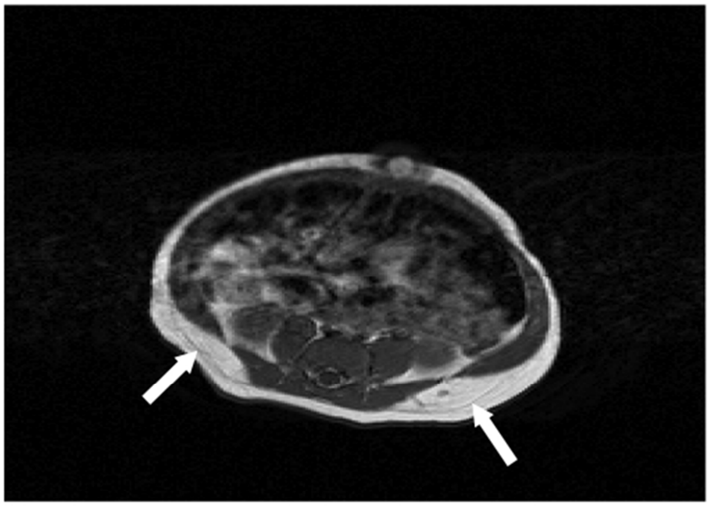

**Figure 2.** T1 weighted axial magnetic resonance image (abdominal level) demonstrating the deep and superficial subcutaneous adipose tissue depots. A clear fascial plane is noted between the superficial and deep subcutaneous layers (arrows).

254x190mm (72 x 72 DPI)

For peer review only

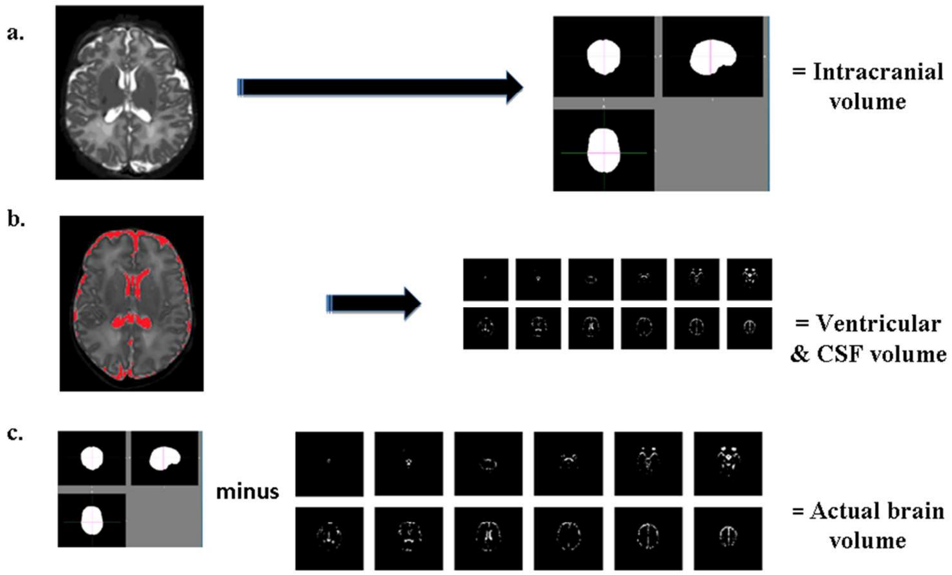


**Figure 3. a.** T2 weighted MR image undergoes RF inhomogeneity correction and subsequent creation of binary brain mask of intracranial volume (BET brain extraction tool FSL version 3). **b.** Creation of a mask of the ventricular and cerebrospinal fluid spaces using the thresholding feature of ImageJ version 1.38. **c.** Calculation of actual brain volume by subtraction of ventricular & CSF volume from intracranial volume.

254x190mm (96 x 96 DPI)

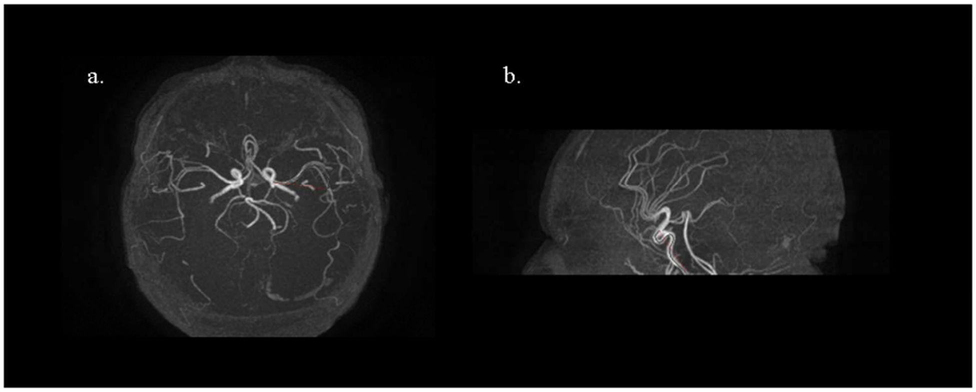


**Figure 4. a.** Axial magnetic resonance angiogram demonstrating tracing of a contour along the left middle cerebral artery for 30mm (black line) along with a straight line measurement between the start point and the end point of the traced contour (red line). **b.** Sagittal magnetic resonance angiogram demonstrating tracing of a contour along the anterior cerebral artery for 30mm (black line) along with a straight line measurement between the start point and the end point of the traced contour (red line).

254x190mm (96 x 96 DPI)

For peer review only

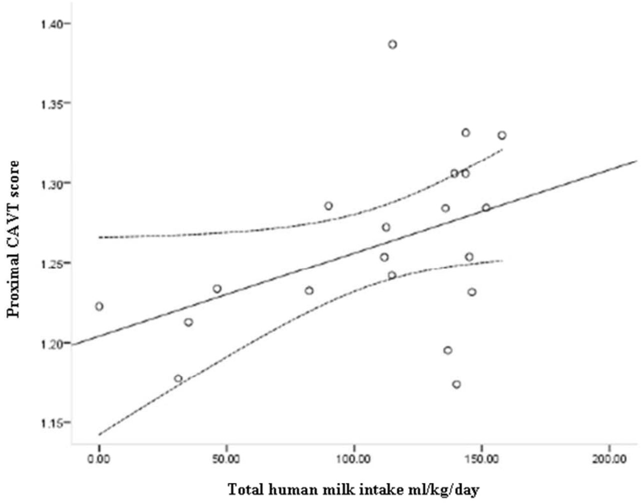


**Figure 5.** Relationship between proximal cerebral arterial vessel tortuosity (CAVT) score at term and total human milk intake (birth to $34^{+6}$ weeks PMA) in preterm infants.

254x190mm (96 x 96 DPI)

STROBE Statement—Checklist of items that should be included in reports of ***cohort studies***

| | Item No | Recommendation |
|---|---|---|
| **Title and abstract** | 1 | (*a*) Indicate the study's design with a commonly used term in the title or the abstract |
| | | (*b*) Provide in the abstract an informative and balanced summary of what was done and what was found |
| **Introduction** | | |
| Background/rationale | 2 | Explain the scientific background and rationale for the investigation being reported |
| Objectives | 3 | State specific objectives, including any prespecified hypotheses |
| **Methods** | | |
| Study design | 4 | Present key elements of study design early in the paper |
| Setting | 5 | Describe the setting, locations, and relevant dates, including periods of recruitment, exposure, follow-up, and data collection |
| Participants | 6 | (*a*) Give the eligibility criteria, and the sources and methods of selection of participants. Describe methods of follow-up |
| | | (*b*) For matched studies, give matching criteria and number of exposed and unexposed |
| Variables | 7 | Clearly define all outcomes, exposures, predictors, potential confounders, and effect modifiers. Give diagnostic criteria, if applicable |
| Data sources/ measurement | 8* | For each variable of interest, give sources of data and details of methods of assessment (measurement). Describe comparability of assessment methods if there is more than one group |
| Bias | 9 | Describe any efforts to address potential sources of bias |
| Study size | 10 | Explain how the study size was arrived at |
| Quantitative variables | 11 | Explain how quantitative variables were handled in the analyses. If applicable, describe which groupings were chosen and why |
| Statistical methods | 12 | (*a*) Describe all statistical methods, including those used to control for confounding |
| | | (*b*) Describe any methods used to examine subgroups and interactions |
| | | (*c*) Explain how missing data were addressed |
| | | (*d*) If applicable, explain how loss to follow-up was addressed |
| | | (*e*) Describe any sensitivity analyses |
| **Results** | | |
| Participants | 13* | (a) Report numbers of individuals at each stage of study—eg numbers potentially eligible, examined for eligibility, confirmed eligible, included in the study, completing follow-up, and analysed |
| | | (b) Give reasons for non-participation at each stage |
| | | (c) Consider use of a flow diagram |
| Descriptive data | 14* | (a) Give characteristics of study participants (eg demographic, clinical, social) and information on exposures and potential confounders |
| | | (b) Indicate number of participants with missing data for each variable of interest |
| | | (c) Summarise follow-up time (eg, average and total amount) |
| Outcome data | 15* | Report numbers of outcome events or summary measures over time |
| Main results | 16 | (*a*) Give unadjusted estimates and, if applicable, confounder-adjusted estimates and their precision (eg, 95% confidence interval). Make clear which confounders were adjusted for and why they were included |
| | | (*b*) Report category boundaries when continuous variables were categorized |
| | | (*c*) If relevant, consider translating estimates of relative risk into absolute risk for a meaningful time period |

For peer review only


For peer review only - http://bmjopen.bmj.com/site/about/guidelines.xhtml

| | | |
|---|---|---|
| Other analyses | 17 | Report other analyses done—eg analyses of subgroups and interactions, and sensitivity analyses |
| **Discussion** | | |
| Key results | 18 | Summarise key results with reference to study objectives |
| Limitations | 19 | Discuss limitations of the study, taking into account sources of potential bias or imprecision. Discuss both direction and magnitude of any potential bias |
| Interpretation | 20 | Give a cautious overall interpretation of results considering objectives, limitations, multiplicity of analyses, results from similar studies, and other relevant evidence |
| Generalisability | 21 | Discuss the generalisability (external validity) of the study results |
| **Other information** | | |
| Funding | 22 | Give the source of funding and the role of the funders for the present study and, if applicable, for the original study on which the present article is based |

*Give information separately for exposed and unexposed groups.

**Note:** An Explanation and Elaboration article discusses each checklist item and gives methodological background and published examples of transparent reporting. The STROBE checklist is best used in conjunction with this article (freely available on the Web sites of PLoS Medicine at http://www.plosmedicine.org/, Annals of Internal Medicine at http://www.annals.org/, and Epidemiology at http://www.epidem.com/). Information on the STROBE Initiative is available at http://www.strobe-statement.org.

For peer review only

BMJ Open

# Preterm nutritional intake and magnetic resonance imaging phenotype at term age: A prospective observational study.

| | |
|---|---|
| Journal: | *BMJ Open* |
| Manuscript ID: | bmjopen-2014-005390.R1 |
| Article Type: | Research |
| Date Submitted by the Author: | 23-Apr-2014 |
| Complete List of Authors: | Vasu, Vimal; William Harvey Hospital, Child Health<br>Durighel, Giuliana; Imperial College, Institute of Clinical Sciences<br>Thomas, E Louise; Imperial College, Metabolic and Molecular Imaging Group<br>Malamateniou, Christina; Kings College, Division of Imaging Sciences<br>Bell, Jimmy; Imperial College London, Metabolic and Molecular Imaging Group<br>Rutherford, Mary; Kings College, Division of Imaging Sciences; Imperial College, MRC Clinical Sciences Centre<br>Modi, Neena; Imperial College London, Neonatal Medicine |
| <b>Primary Subject Heading</b>: | Paediatrics |
| Secondary Subject Heading: | Nutrition and metabolism, Paediatrics, Radiology and imaging |
| Keywords: | NEONATOLOGY, Neonatal intensive & critical care < INTENSIVE & CRITICAL CARE, Magnetic resonance imaging < RADIOLOGY & IMAGING |
| | |

only

SCHOLARONE™
Manuscripts

For peer review only - http://bmjopen.bmj.com/site/about/guidelines.xhtml

**Title:** Preterm nutritional intake and magnetic resonance imaging phenotype at term age: A prospective observational study.

**Authors:** Vimal Vasu[1,5] MBBS MD, Giuliana Durighel[2] MSc, Louise Thomas[2] PhD, Christina Malamateniou[3,4] PhD, Jimmy D Bell[2] PhD, Mary A Rutherford[3,4] MBBS MD, Neena Modi[1] MBChB MD

**Affiliations:** [1]Section of Neonatal Medicine, Department of Medicine, Imperial College London, Chelsea & Westminster Hospital campus, 369 Fulham Road, London, SW10 9NH, UK; [2]Metabolic and Molecular Imaging Group, MRC Clinical Sciences Centre, Imperial College London, London, W12 0HS, UK; [3]Division of Clinical Sciences, Imperial College London, MRC Clinical Sciences Centre Hammersmith Hospital, Du Cane Road, London, United Kingdom, W12 0HS, UK; [4] Centre for the Developing Brain, Department of Perinatal Imaging & Health, Division of Imaging Sciences & Biomedical Engineering, Faculty of Medicine, King's College London, St Thomas' Hospital, London SE1 7EH, UK; [5] Department of Child Health, William Harvey Hospital, Ashford, Kent, TN24 0LZ, UK.

**Address Correspondence to:** Dr Vimal Vasu, Department of Child Health, William Harvey Hospital, Ashford, Kent, TN24 0LZ, United Kingdom.

Email: vimal.vasu@nhs.net

**Short Title:** Preterm nutrition and phenotype

For peer review only

**Abbreviations:** ACA-anterior cerebral artery, AT-adipose tissue, CAVT-cerebral arterial vessel tortuosity, CRIB II-Clinical Risk Index for Babies II, CSF-Cerebrospinal fluid, DSC-deep subcutaneous, I-internal, IHCL-intrahepatocellular lipid, MCA-middle cerebral artery, MR-magnetic resonance, NMR-nuclear magnetic resonance, PMA-post menstrual age, PCA-posterior cerebral artery, SDS- standard deviation scores, SSC-superficial subcutaneous

**Key Words:** preterm, nutrition, body composition, magnetic resonance

For peer review only

## ABSTRACT

**Objective** To describe (i) the relationship between nutrition and the preterm-at-term infant phenotype, (ii) phenotypic differences between preterm-at-term infants and healthy term born infants and (iii) relationships between somatic and brain MRI outcomes.

**Design** Prospective observational study.

**Setting** UK tertiary neonatal unit.

**Participants** Preterm infants (<32 weeks gestation) (n=22) and healthy term infants (n=39)

**Main outcome measures** Preterm nutrient intake; total and regional adipose tissue (AT) depot volumes; brain volume and proximal cerebral arterial vessel tortuosity (CAVT) in preterm infants and in term infants.

**Results** Preterm nutrition was deficient in protein, high in carbohydrate and high in fat. Preterm nutrition was not related to AT volumes, brain volume or proximal CAVT score; a positive association was noted between human milk intake and proximal CAVT score ($r=0.44$, $p=0.05$). In comparison to term infants, preterm infants had increased total adiposity, comparable brain volumes and reduced proximal CAVT scores. There was a significant negative correlation between deep subcutaneous abdominal adipose tissue volume and brain volume in preterm infants ($r= -0.58$, $p=0.01$).

**Conclusions** Though there are significant phenotypic differences between preterm infants at term and term infants, preterm macronutrient intake does not appear to be a determinant. Our preliminary data suggest that (i) human milk maybe exert a beneficial effect upon cerebral arterial vessel tortuosity (ii) there is a negative correlation between adiposity and brain volume in preterm infants at term. Further


For peer review only

work is warranted to see if our findings can be replicated and to understand the causal mechanisms.

For peer review only

**ARTICLE SUMMARY**

- Provision of nutrition to preterm infants is complicated by immaturity, critical illness, and a limited evidence base relating early nutrition to functional health outcomes.
- In comparison to term infants, preterm infant phenotype at term is characterized by adiposity, comparable brain volume, increased CSF volume and reduced cerebral artery tortuosity.
- Though preterm macronutrient intake is not a determinant of this phenotype, human milk intake is positively associated with cerebral artery tortuosity.
- In preterm infants, regional adiposity is negatively correlated with brain volume measured at term age.

For peer review only

**Strengths of study**

- No previously published studies have studied the relationship between preterm nutritional intake and MRI outcomes at term age.
- Comprehensive ascertainment of preterm nutritional data in parallel with somatic and brain MRI.
- Use of term born infants as comparator for MRI outcomes.

**Limitations of study**

- Limited sample size.
- Prospective observational nature of study.

For peer review only

## INTRODUCTION

Preterm nutritional guidelines are based upon consensus expert opinion (1,2) rather than compelling evidence and the "optimal" diet for long term health remains unknown (3). Though there is now greater clinical emphasis upon preterm nutrition, protein deficiency remains a significant risk and our previously published data in 18 preterm infants from a single centre indicate that the preterm diet is low in protein whilst being high in both carbohydrate and fat (4). Our data also suggest that preterm macronutrition may affect later health by demonstrating a positive association between first week lipid intake in preterm infants and elevated intrahepatocellular lipid (IHCL), which in adults is associated with the cardio-metabolic syndrome (5).

The preterm phenotype at term is characterized by aberrant adipose tissue partitioning (6), reduced proximal cerebral arterial vessel tortuosity (CAVT) (7), reduced deep grey matter volumes (8), and reduced cerebral cortical folding (9). The somatic phenotype observed is of concern as adiposity is associated with inflammation and reduced brain volume in the adult population (10).

Here we present prospective observational data designed to (i) assess the influence of nutrition upon the preterm phenotype at term age (ii) describe phenotypic differences between preterm infants at term and term healthy infants and (iii) examine relationships between somatic and brain MRI measurements. The a priori hypotheses of our study were that (i) preterm macronutrient intake would be positively associated with central nervous system phenotype (brain volume & cerebral vessel tortuosity) and (ii) preterm macronutrient intake would be negatively associated with internal

For peer review only

abdominal (visceral) adiposity. The relationship between somatic and brain MRI outcomes represents a post hoc exploratory analysis.

For peer review only

## PATIENTS AND METHODS

Following research ethics approval (REC 07/Q0403/46) and informed parent consent, preterm infants admitted to Chelsea and Westminster Hospital neonatal unit, London, UK (< 32 weeks of gestation) and term infants (37-42 weeks gestation) on the postnatal ward were recruited (January 2007-July 2008). Infants with congenital anomalies were excluded.

### Preterm nutrition and growth

Preterm nutritional practice during the study period has been previously described (4). In brief, enteral feeds were commenced on day 1 with either maternal expressed breast milk or donor expressed breast milk prior to consideration of the use of formula. Human milk fed infants received Nutriprem breast milk fortifier (Cow & Gate) once 150ml/kg/day of feed volume was reached. Parenteral nutrition was commenced on the day of birth.

Weight and head circumference data for the preterm group are expressed as SDS at birth and at time of MRI. Birth length was not routinely measured during the study period. Macronutrient and human milk intake was recorded between birth and $34^{+6}$ weeks PMA using a nutritional data capture system designed in-house (Nutcracker™, Imperial College, London, UK). Macronutrient data were expressed as the difference between recommended daily intake (RDI) from Tsang et al (i) and actual daily intake in g or kcal/kg for the period of either early (first week of life) or total nutrition (birth and $34^{+6}$ PMA) (iv). Human milk intake is expressed as ml/kg/day and as percentage of enteral feeds given as human milk.

**Magnetic Resonance Imaging**

MRI was performed at the Robert Steiner Unit, Hammersmith Hospital shortly after discharge from the neonatal unit as previously described (iv) using a Philips Achieva system (Best, the Netherlands).

*(i) Total and Regional AT*

Imaging parameters are shown in Table 1. Regional AT depots were classified as total superficial subcutaneous, total deep subcutaneous and total internal and subdivided into abdominal or non-abdominal (Figures 1 & 2). AT volumes were calculated as previously described (6). Non AT mass was calculated by conversion of AT volume to AT mass (density of AT of 0.9 grams/$cm^3$) and subtraction of the result from weight at time of MRI.

*(ii) Brain volume*

T2 weighted images were acquired using a dynamic sequence of six separate loops of single shot images which were then registered and reconstructed to produce volumetric datasets to eliminate the effects of motion artefact (11). Imaging parameters are shown in Table 1. Images were corrected for Radiofrequency inhomogeneity (http://mipav.cit.nih.gov). BET Brain extraction tool FSL Version 3 (http://www.fmrib.ox.ac.uk/fsl/) (12) was then used to delete non brain tissue and create binary brain masks representing intracranial volume (13). A mask of ventricular and CSF spaces was created using the thresholding feature of Image J, a java based image processing program (14). Brain volume was calculated by subtraction of the volume of the ventricular and CSF mask from the volume of the intracranial mask using ImageJ (Figure 3). Brain MRIs were reported by MR for

For peer review only - http://bmjopen.bmj.com/site/about/guidelines.xhtml

clinical purposes and given a score (0-13) adapted from Dyet et al (15) so that if necessary, pathological findings could be accounted for.

*(iii) Proximal cerebral arterial vessel tortuosity (CAVT) measurement and analysis*

An optimized neonatal three dimensional time of flight MRA sequence was used to assess the anterior, middle and posterior cerebral arteries (7). Imaging parameters are shown in Table 1. Vessel tortuosity was assessed using a previously validated measurement, distance factor (16) (Figure 4) and a CAVT score was determined as a global measure of tortuosity for each subject by calculating the mean of the ACA, MCA and PCA distance factor.

**Illness severity**

CRIB II Score was calculated in preterm infants (17).

**Sample size calculation and analyses**

Based on previous work (6), we estimated that recruitment of 60 PT and 60 T infants allowed detection of a 0.5 SD difference between the groups for AT volume (Power 80%, Significance 5%). As this was an exploratory hypothesis-generating prospective observational study and given the uncertainty as to what differences were of clinical importance, additional sample size calculations were not considered. Data were adjusted for relevant confounding variables where appropriate and are presented for each of the MR outcomes with a comparison of outcomes between the groups. Data were analyzed by comparison of means and tested for normality. Parametric or non parametric methods were then applied accordingly. Within the preterm group, Pearson Correlation was used to investigate the relationship between nutrition and MR

For peer review only

outcomes at term age and these were limited to the patients in who both nutritional data and the MRI outcome were successfully acquired. For a multivariate analysis a minimum of 10n subjects is considered appropriate, where n is the number of covariates. Data are presented as mean (SD) or mean (95% CI).

For peer review only

## RESULTS

61 infants were recruited during the study (Preterm 22, Term 39). MR images without motion artefact were acquired as follows: AT volume (preterm 22, term 39), brain volume (preterm 19, term 19), CAVT measurement (preterm 20, term 13). In preterm infants, mean (SD) CRIB II score was 7.6 (4.2). 25% had chronic lung disease of prematurity (defined as an oxygen requirement at 36 weeks PMA), 20% had a patent ductus arteriosus requiring pharmacological therapy, 5% had retinopathy of prematurity requiring laser therapy and 5% had a large intraventricular haemorrhage (Grade III/IV Papile classification). 0% had necrotising enterocolitis requiring surgery. Table 2 summarises the baseline and imaging characteristics and the MR outcomes of the preterm and term groups.

### Preterm nutrition & growth

Growth and nutrition data for the preterm cohort have been previously published (4). In brief, mean (SD) for birth weight and birth head circumference SDS were: -0.13 (0.78) and -0.64 (1.12). At time of MRI, the respective values were: -1.39 (0.93) and -0.86 (1.33). Weight gain was mean (SD) 9.48 (1.73) g/kg/day between birth and $34^{+6}$ weeks. Preterm macronutrient intakes for both the first week after birth and for the period from birth until $34^{+6}$ weeks post menstrual age revealed a mean protein deficit (first week: -1.6g/kg/day; birth until $34^{+6}$ weeks -0.4g/kg/day) in the context of excessive carbohydrate and fat intake. Mean (SD) human milk intakes for these periods were 36.6 (29.7) ml/kg/day and 108.9 (46.6) ml/kg/day respectively.

**Preterm Nutrition and MRI Outcomes**

*(i) Human milk and MRI outcomes*

After adjustment for weight at MRI, there was no correlation between early or total human milk intake and either regional AT volumes or brain volume at term age. There was no correlation between early human milk intake and overall CAVT score (r= 0.31, p=0.18); however, there was a weak positive relationship between total human milk intake and overall CAVT score (r=0.44, p=0.05) (Figure 5). This relationship was also apparent when total human milk intake was expressed as percentage of total milk intake (r=0.45, p=0.04).

*(ii) Macronutrient intake and MRI outcomes*

There were no correlations between macronutrient intake (protein, carbohydrate, fat) and total adiposity (n=22). After adjustment for PMA, neither early nor total preterm macronutrient intake were correlated with brain volume at term age (n=19). There were no relationships noted between early or total macronutrient intake and proximal CAVT score (n=20), a summary measure of tortuosity.

**Preterm at Term versus Term Group Comparison**

*(i) Anthropometry*

Anthropometric data were adjusted for PMA at time of MRI. Preterm infants were smaller, shorter and had smaller head circumferences than term infants (Table 2).

For peer review only

*(ii) Adiposity*

Adipose tissue data were adjusted for PMA at time of MRI. Preterm infants had more AT than term infants with expansion of the superficial subcutaneous, deep subcutaneous and internal AT depots. There was a parallel reduction in non adipose tissue mass (Table 2).

*(iii) Brain volume*

Brain MRI scores in the cohort of preterm infants were mean (SD) 3.6 (2.1). There was no correlation between brain MRI score and brain volume (r=0.31, *p*=0.20). At term age, preterm brain volumes were smaller than term infants [PT 461.74 (436.16-487.32) ml v 493.74 (476.43-511.05) ml, *p*=0.05. However, after adjustment for weight at time of MRI, a significant confounding factor, there was no difference between the groups. Preterm-at-term infants had significantly increased CSF volume (Table 2).

*(iv) Distance Factor & Proximal CAVT score*

In comparison to term born infants, preterm-at-term infants had a significant reduction in cerebral arterial vessel tortuosity (Table 2).

**Relationships between somatic and central nervous system MRI phenotype**

After adjustment for weight at MRI, there was a significant negative correlation noted in preterm at term infants but not in term infants, between deep subcutaneous AT volume and brain volume (r=-0.58, *p*=0.01) (Table 3). There were no statistically significant correlations between regional AT and CAVT score.

For peer review only

**DISCUSSION**

We identify novel findings of interest including a positive association between human milk intake and proximal CAVT, a marker of cerebrovascular development and a negative correlation between regional adipose tissue volume and brain volume in preterm infants. The study also comprehensively characterises the somatic and brain phenotype of a cohort of preterm infants at term in comparison to term born healthy infants and demonstrates (i) reduced anthropometric measures (ii) increased total and regional adiposity (iii) reduced non-adipose tissue mass (iv) comparable brain volumes (v) increased CSF volume and (vi) reduced proximal cerebral arterial vessel tortuosity. We have shown no relationship with either body composition or brain volume at term age within the range of macronutritional intakes received by the preterm infants in this study.

Key study strengths include the comprehensive ascertainment of preterm nutritional data and the assessment of a number of MR outcomes. Limitations include the prospective observational design of the study that preclude any inferences regarding causality, and the “sub-optimal” preterm nutritional intake, a potential determinant of the observed phenotype. Successful acquisition of a number of different MR outcomes without use of sedation was challenging and motion artefact meant that not all recruited infants had data of sufficient quality for analysis. We were unable to recruit the desired number of infants and recognise that the study may be underpowered. The preterm infants studied were relatively healthy, as evidenced by low illness severity (CRIB II scores), low incidences of serious neonatal morbidity and low brain MR scores, factors that may have attenuated any associations with nutritional intake.

For peer review only

The finding of increased adiposity in preterm infants confirms our previous work (6). We have previously shown expansion of the internal abdominal AT compartment in preterm at term infants in comparison to term born infants in a cohort of preterm infants recruited in 2002-2003 (6), a time at which neonatal nutrition was possibly not as carefully considered as it is today. This contrasts with our present data which demonstrates a global expansion of all AT depots. Whether the differences observed in AT partitioning between these studies relate to changes in nutritional practises and the provision of a more calorie dense diet is a plausible but as yet unproven hypothesis. Other potential mechanisms, which we have not explored, that might explain the increased adiposity seen in preterm infants include weight cycling and inflammation. It is known that cycling between high and low calorie diets (weight cycling) (18) results in the preferential deposition of AT over non AT mass (19) and this phenomenon often occurs in preterm infants when enteral feeds are discontinued because of concerns regarding feed intolerance and then restarted. Inflammation is also a known determinant of adiposity (20) and it is possible that the pro-inflammatory milieu often present in the perinatal period (maternal chorioamnionitis, use of intravenous lipid formulations high in omega-6 fatty acids and postnatal infection/ inflammation) may be relevant to the observed phenotype.

For peer review only

The nutritional intake received by this cohort of preterm infants was imbalanced (low in protein and high in both fat and carbohydrate) and sub-optimal in relation to expert consensus recommendations (1,2). In animal models, protein deficiency is associated with a number of adverse health outcomes including a reduction in life span (21), cardiovascular dysfunction (22), reduced dendritic spine density (23), reduced brain

weight (24) and reduced cortical blood vessel density (25). Human adult data indicate that dietary protein is an important factor in body weight regulation (26). Recent data in human ex-preterm infants suggests an association between early growth patterns and fractional anisotropy, a measure of brain microstructure (27). Our group have previously shown that preterm at term AT deposition can be attenuated by use of fortified human milk (28). Whether this translates into longer term benefit is unknown.

The negative correlation we show between adiposity in preterm at term infants and brain volume is consistent with findings in adults and children (10,29,30). This notwithstanding preterm at term brain volume was comparable to that of term born healthy infants which is also in keeping with previously published work (31). This, together with the finding of maintained head circumference SDS between birth and time of imaging may be indicative of "brain sparing" in nutritionally compromised infants.

We have confirmed the finding of reduced proximal CAVT in preterm infants (7). Though the natural history and long term neurodevelopmental sequelae of reduced CAVT are unknown, epidemiological data indicate that advancing gestation confers a significant reduction in risk of fatal adult cerebrovascular disease (occlusive stroke) (32). Our observation that human milk may be protective despite low macronutrient density, suggests that non-nutritive factors, such as vascular endothelial growth factor may play an important role in cerebrovascular development.

In conclusion, we have extended the characterization of the preterm-at-term phenotype. Our data do not support an association between macronutrient intake and body composition or brain volume. Other plausible determinants that remain to be explored are the roles of micronutrient deficiency, weight cycling, disease severity, and chronic inflammation.

For peer review only

## FIGURE LEGENDS

**Figure 1.** Classification of adipose tissue (AT) depots into internal, superficial subcutaneous and deep subcutaneous depots and further sub-classification according to abdominal or non abdominal position.

**Figure 2.** T1 weighted axial magnetic resonance image (abdominal level) demonstrating the deep and superficial subcutaneous adipose tissue depots. A clear fascial plane is noted between the superficial and deep subcutaneous layers (arrows).

**Figure 3.** a. T2 weighted MR image undergoes RF inhomogeneity correction and subsequent creation of binary brain mask of intracranial volume (BET brain extraction tool FSL version 3). b. Creation of a mask of the ventricular and cerebrospinal fluid spaces using the thresholding feature of ImageJ version 1.38. c. Calculation of actual brain volume by subtraction of ventricular & CSF volume from intracranial volume.

**Figure 4.** a. Axial magnetic resonance angiogram demonstrating tracing of a contour along the left middle cerebral artery for 30mm (black line) along with a straight line measurement between the start point and the end point of the traced contour (red line). b. Sagittal magnetic resonance angiogram demonstrating tracing of a contour along the anterior cerebral artery for 30mm (black line) along with a straight line measurement between the start point and the end point of the traced contour (red line).

For peer review only

**Figure 5.** Relationship between proximal cerebral arterial vessel tortuosity (CAVT) score at term and total human milk intake (birth to $34^{+6}$ weeks PMA) in preterm infants.

For peer review only

**Table 1.** MR imaging parameters for adipose tissue, brain volume and proximal cerebral arterial vessel tortuosity.

| | Planes | Weighting | FOV | Slices | Slice thickness | Slice gap | TE | TR | Acquisition time |
|---|---|---|---|---|---|---|---|---|---|
| **Adipose tissue**[6] | Axial | T1 Fast Spin Echo | 300 | 25 | 5mm | 5mm | 20ms | 500ms | 360s |
| **Brain volume**[11] | Axial/Sagittal | T2 | 220 | 60 | 3mm | -1.5mm | 160ms | 16646/38662ms | 425s |
| **Proximal cerebral arterial vessel tortuosity**[7] | Axial/Sagittal | Angiogram | 175 | 100 | 0.6mm | 0mm | 8.06ms | 19ms | 329s |

For peer review only

**Table 2.** Birth characteristics, imaging characteristics and MRI outcomes in preterm-at-term and term infants.

| | Preterm-at-Term | n | Term | n | 95% CI for Difference |
|---|---|---|---|---|---|
| **Birth Characteristics** | | | | | |
| Gestation at Birth (weeks) | 28.9 (2.8) | 22 | 40.2 (1.20) | 39 | N/A |
| Birth Weight (kg) | 1.26 (0.41) | 22 | 3.40 (0.45) | 39 | N/A |
| Gestation at MRI (weeks) | 40.2 (3.10) | 22 | 41.4 (1.40) | 39 | N/A |
| | | | | | |
| **Anthropometry at time of MRI** | | | | | |
| Weight at MRI (kg) | 3.01 (2.83-3.19) | 22 | 3.30 (3.17-3.44) | 39 | 0.30 (0.66-0.53)* |
| Length at MRI (cm) | 47.9 (46.9-48.9) | 22 | 51.3 (50.5-52.1) | 39 | 3.4 (2.1-4.7)** |
| Head Circumference at MRI (cm) | 34.2 (33.6-34.7) | 22 | 35.2 (34.8-35.6) | 39 | 1.1 (0.4-1.7)** |
| | | | | | |
| **Somatic MRI Outcomes** | | | | | |
| Total AT (l) | 0.782 (0.735-0.829) | 22 | 0.657 (0.623-692) | 39 | 0.125 (0.064-0.186)** |
| Total Deep Subcutaneous AT (l) | 0.033 (0.029-0.036) | 22 | 0.024 (0.021-0.027) | 39 | 0.009 (0.004-0.013)** |
| Total Superficial Subcutaneous AT (l) | 0.673 (0.631-0.715) | 22 | 0.568 (0.538-0.599) | 39 | 0.105 (0.051-0.159)** |
| Total Internal AT (l) | 0.076 (0.069-0.083) | 22 | 0.065 (0.060-0.070) | 39 | 0.011 (0.002-0.020)* |
| Deep Subcutaneous Adipose Abdominal AT (l) | 0.017 (0.014-0.019) | 22 | 0.012 (0.011-0.014) | 39 | 0.004 (0.001-0.005)** |
| Deep Subcutaneous Adipose Non Abdominal AT (l) | 0.016 (0.014-0.018) | 22 | 0.012 (0.010-0.013) | 39 | 0.004 (0.002-0.007)** |
| Superficial Subcutaneous Adipose Abdominal AT (l) | 0.133 (0.122-0.145) | 22 | 0.091 (0.083-0.100) | 39 | 0.042 (0.027-0.057)** |
| Superficial Subcutaneous Adipose Non Abdominal AT (l) | 0.540 (0.506-0.574) | 22 | 0.477 (0.452-0.502) | 39 | 0.063 (0.019-0.107)** |
| Internal Abdominal AT (l) | 0.022 (0.019-0.025) | 22 | 0.018 (0.016-0.021) | 39 | 0.004 (0-0.007) |
| Internal Non Abdominal AT (1) | 0.054 (0.049-0.060) | 22 | 0.047 (0.043-0.051) | 39 | 0.008 (0.001-0.005)* |
| Non AT mass (kg) | 2.49 (2.45-2.54) | 22 | 2.61 (2.57-2.64) | 39 | 0.12 (0.06-0.170)** |
| | | | | | |
| **Brain MRI Outcomes** | | | | | |
| Brain Volume (ml) | 481.46 (462.29-498.63) | 19 | 474.02 (456.85-491.20) | 19 | 7.44 (18.53-33.40) |
| Cerebrospinal Fluid Volume (ml) | 63.78 (55.06-72.50) | 19 | 31.44 (22.72-40.16) | 19 | 32.34 (19.15-45.53)** |
| Anterior Cerebral Artery DF | 1.38 (1.30-1.46) | 20 | 1.25 (1.19-1.32) | 13 | 0.13 (0.03-0.23)* |
| Middle Cerebral Artery DF | 1.38 (1.32-1.44) | 20 | 1.26 (1.21-1.31) | 13 | 0.12 (0.04-0.19)** |
| Posterior Cerebral Artery DF | 1.46 (1.41-1.50) | 20 | 1.27 (1.24-1.30) | 13 | 0.19 (0.14-0.24)** |
| Proximal CAVT score | 1.41 (1.36-1.45) | 20 | 1.26 (1.23-1.29) | 13 | 0.14 (0.09-0.20)** |

*Footnotes: Results are mean (SD) or mean (95% CI). * p<0.05, **p<0.01. (AT-adipose tissue, DF- distance factor, CAVT- cerebral arterial vessel tortuosity, n- sample size).*

For peer review only

**Table 3.** Pearson correlations between regional adipose tissue (AT) depots and brain volume in preterm at term and term infants adjusted for weight at imaging.

| Regional AT depot | Preterm at Term (n=19) | Term (n=19) |
|---|---|---|
| Total AT | -0.23, p=0.38 | -0.23, p=0.35 |
| Total DSC AT | -0.58, p=0.01* | -0.05, p=0.85 |
| Total SSC AT | -0.22, p=0.39 | -0.24, p=0.34 |
| Total I AT | -0.09, p=0.73 | -0.13, p=0.96 |

*AT: Total adipose tissue, Total I AT:Total internal adipose tissue, Total SSC AT:Total superficial subcutaneous adipose tissue, Total DSC AT:Total deep subcutaneous adipose tissue, IA AT:Internal abdominal adipose tissue.*

For peer review only

**Funding Sources:** United Kingdom Medical Research Council; Chelsea and Westminster Hospital NHS Foundation Trust

**Financial Disclosure:** None

**Conflict of Interest:** None

**Clinical Trials Registration:** Not Applicable

**Data sharing statement:** No additional data available

**Contributorship Statement:** VV helped design the original prospective study, analysed the brain MRI data, drafted the initial manuscript and approved the final manuscript as submitted. GD optimized MR imaging parameters for the study. LT coordinated analysis of the adipose tissue MRI data. CM optimised MR angiography and advised on vessel tortuosity measurements. JB was involved in study design and supervised analysis of the adipose tissue MRI data. MR was involved in study design and supervised analysis of brain MRI data. NM was involved with study design and supervised the analysis of neonatal nutritional data. All authors critically reviewed and revised the manuscript and approved the final manuscript as submitted.

For peer review only

## REFERENCES

1. Tsang R, Uauy R, Koletzko B et al. Nutrition of the preterm infant: Scientific basis and practical guidelines. 2nd ed. Cincinnati, OH: Digital Education Publishing, Inc; 2005: 415-416.

2. Agostoni C, Buonocore G, Carnielli VP et al. Enteral nutrient supply for preterm infants: commentary from the European Society of Paediatric Gastroenterology, Hepatology and Nutrition Committee on Nutrition. *J Pediatr Gastroenterol Nutr*. 2010;50(1):85-91.

3. Vasu V, Modi N. Assessing the impact of preterm nutrition. *Early Hum Dev*. 2007;83(12):813-818.

4. Vasu V, Thomas EL, Durighel G et al. Early Nutritional Determinants of Intrahepatocellular Lipid Deposition in Preterm Infants at Term Age. *Int J Obes (Lond)*. 2013;37(4);500-504.

5. Mehta SR, Godsland IF, Thomas EL et al. Intrahepatic insulin exposure, intrahepatocellular lipid and regional body fat in nonalcoholic fatty liver disease. *J Clin Endocrinol Metab*. 2012;97(6):2151-2159.

6. Uthaya S, Thomas EL, Hamilton G et al. Altered adiposity after extremely preterm birth. *Pediatr Res*. 2005;57(2):221-225.

For peer review only

7. Malamateniou C, Counsell SJ, Allsop JM et al. The effect of preterm birth on neonatal cerebral vasculature studied with magnetic resonance angiography at 3 Tesla. *Neuroimage*. 2006;32(3):1050-1059.

8. Srinivasan L, Dutta R, Counsell SJ et al. Quantification of deep gray matter in preterm infants at term-equivalent age using manual volumetry of 3-tesla magnetic resonance images. *Pediatrics*. 2007;119:759-765.

9. Ajayi-Obe M, Saeed N, Cowan FM et al. Reduced development of cerebral cortex in extremely preterm infants. *Lancet*. 2000;356(9236):1162-1163.

10. Debette S, Wolf C, Lambert JC et al. Abdominal obesity and lower gray matter volume: a Mendelian randomization study. Neurobiology of Aging. 2014;35(2):378-386. Epub 2013 Aug 31.

11. Jiang S, Xue H, Glover A et al. MRI of moving subjects using multislice snapshot images with volume reconstruction (SVR): application to fetal, neonatal, and adult brain studies. *IEEE Trans Med Imaging*. 2007;26(7):967-980.

12. Smith SM, Jenkinson M, Woolrich MW et al. Advances in functional and structural MR image analysis and implementation as FSL. *Neuroimage*. 2004;23 Suppl 1:S208-219.

13. Smith SM. Fast robust automated brain extraction. *Hum Brain Mapp*. 2002;17(3):143-155.

For peer review only

14. Rasband, W.S. ImageJ, U. S. National Institutes of Health, Bethesda, Maryland, USA, http://imagej.nih.gov/ij/, 1997-2012.

15. Dyet LE, Kennea N, Counsell SJ et al. Natural History of Brain Lesions in Extremely Preterm Infants Studied With Serial Magnetic Resonance Imaging From Birth and Neurodevelopmental Assessment. *Pediatrics*. 2006;118:536 - 548.

16. Bullitt E, Gerig G, Pizer SM et al. Measuring tortuosity of the intracerebral vasculature from MRA images. *IEEE Trans Med Imaging*. 2003;22(9):1163-1171.

17. Parry G, Tucker J, Tarnow-Mordi W. CRIB II : an update of the Clinical Risk Index for Babies score. *Lancet*. 2003;361:1789-1791.

18. Brownell KD, Greenwood MR, Stellar E et al. The effects of repeated cycles of weight loss and regain in rats. *Physiol Behav*. 1986;38(4):459-464.

19. Rodin J, Radke-Sharpe N, Rebuffé-Scrive M et al. Weight cycling and fat distribution. *Int J Obes*. 1990;14:303-310.

20. So PW, Herlihy AH, Bell JD. Adiposity induced by adenovirus inoculation. *Int J Obes (Lond)*. 2005;29(6):603-606.

For peer review only

21. Ozanne SE, Hales CN. Lifespan: Catch-up growth and obesity in male mice. *Nature*. 2004;427(6973):411-412.

22. Cleal JK, Poore KR, Boullin JP et al. Mismatched pre- and postnatal nutrition leads to cardiovascular dysfunction and altered renal function in adulthood. *PNAS*. 2007;104(22):9529-9533.

23. Garcia-Ruiz M, az-Cintra S, Cintra L et al. Effect of protein malnutrition on CA3 hippocampal pyramidal cells in rats of three ages. *Brain Res*. 1993;625(2):203-12.

24. Perez-Torrero E, Torrero C, Salas M. Effects of perinatal undernourishment on neuronal development of the facial motor nucleus in the rat. *Brain Res*. 2001;905:54-62.

25. Bennis-Taleb N, Remacle C, Hoet JJ et al. A Low-Protein Diet During Gestation Affects Brain Development and Alters Permanently Cerebral Cortex Blood Vessels in Rat Offspring. *J Nutr*. 1999;129(8):1613-1619.

26. Westerterp-Plantenga MS, Lejeune MP, Nijs I et al. High protein intake sustains weight maintenance after body weight loss in humans. *Int J Obes Relat Metab Disord*. 2004:28(1):57–64.

27. Vinall J, Grunau RE, Brant R et al. Slower postnatal growth is associated with delayed cerebral cortical maturation in preterm newborns. *Sci Transl Med*. 2013;5(168):168ra8.

For peer review only

28. Uthaya S, Thomas EL, Bell J et al. Improved growth and adipose tissue partitioning in preterm infants receiving exclusive enteral feeds of fortified human milk in comparison with infants receiving human milk and formula.Proceedings of the Neonatal Society. 2007. (http://www.neonatalsociety.ac.uk/abstracts/uthayas_2007_growthfortifiedfeeds.shtml - 7KB)

29. Yau PL, Castro MG, Tagani A et al. Obesity and metabolic syndrome and functional and structural brain impairements in adolescence. Pediatrics. 2012;130(4):e856-864.

30. Debette S, Beiser A, Hoffmann U et al. Visceral fat is associated with lower brain volume in healthy middle-aged adults. *Ann Neurol*. 2010;68(2):136-144.

31. Boardman JP, Counsell SJ, Rueckert D et al. Early growth in brain volume is preserved in the majority of preterm infants. *Ann Neurol*. 2007;62(2):185-192

32. Koupil I, Leon DA, Lithell HO. Length of gestation is associated with mortality from cerebrovascular disease. *J Epidemiol Community Health*. 2005;59:473-474.

For peer review only

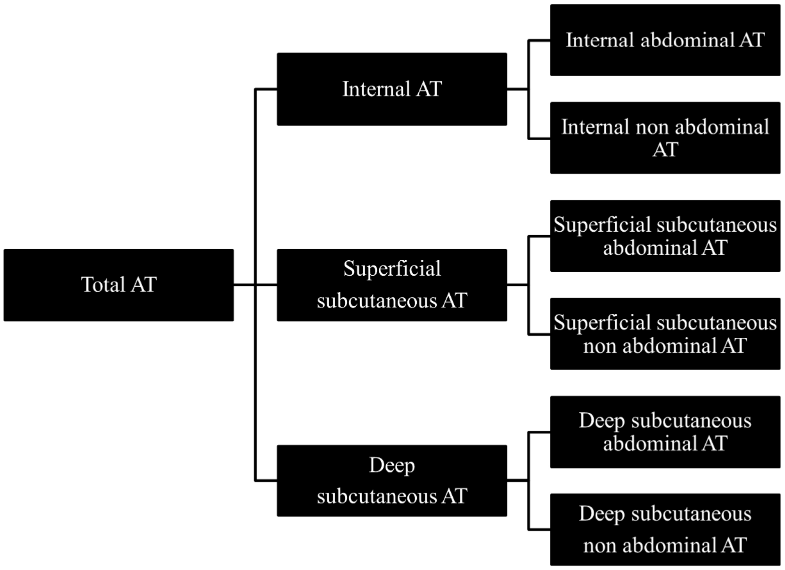


**Figure 1.** Classification of adipose tissue (AT) depots into internal, superficial subcutaneous and deep subcutaneous depots and further sub-classification according to abdominal or non abdominal position.

For peer review only

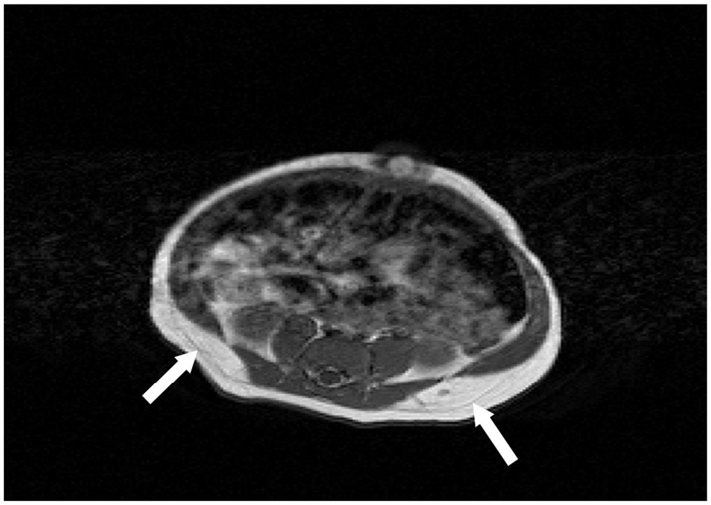

**Figure 2.** T1 weighted axial magnetic resonance image (abdominal level) demonstrating the deep and superficial subcutaneous adipose tissue depots. A clear fascial plane is noted between the superficial and deep subcutaneous layers (arrows).

For peer review only

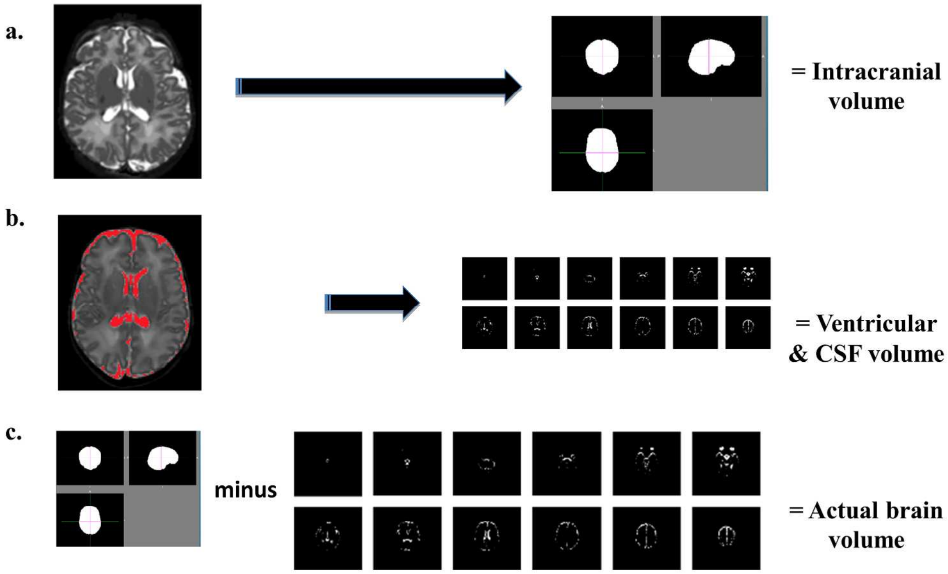


**Figure 3. a.** T2 weighted MR image undergoes RF inhomogeneity correction and subsequent creation of binary brain mask of intracranial volume (BET brain extraction tool FSL version 3). **b.** Creation of a mask of the ventricular and cerebrospinal fluid spaces using the thresholding feature of ImageJ version 1.38. **c.** Calculation of actual brain volume by subtraction of ventricular & CSF volume from intracranial volume.

For peer review only

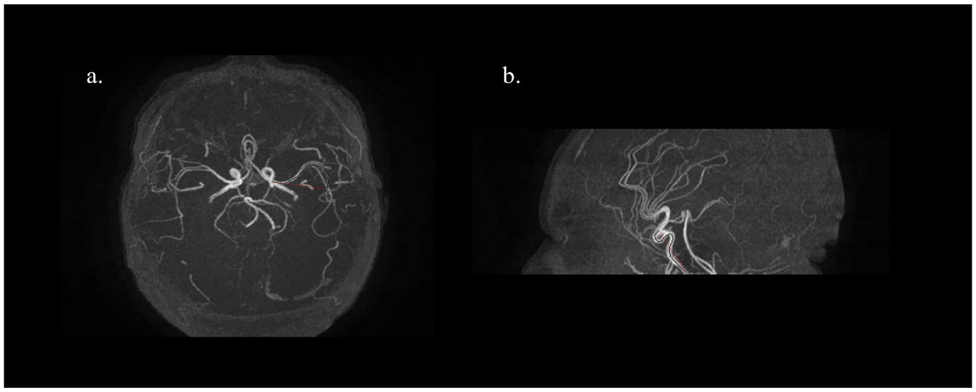


**Figure 4. a.** Axial magnetic resonance angiogram demonstrating tracing of a contour along the left middle cerebral artery for 30mm (black line) along with a straight line measurement between the start point and the end point of the traced contour (red line). **b.** Sagittal magnetic resonance angiogram demonstrating tracing of a contour along the anterior cerebral artery for 30mm (black line) along with a straight line measurement between the start point and the end point of the traced contour (red line).

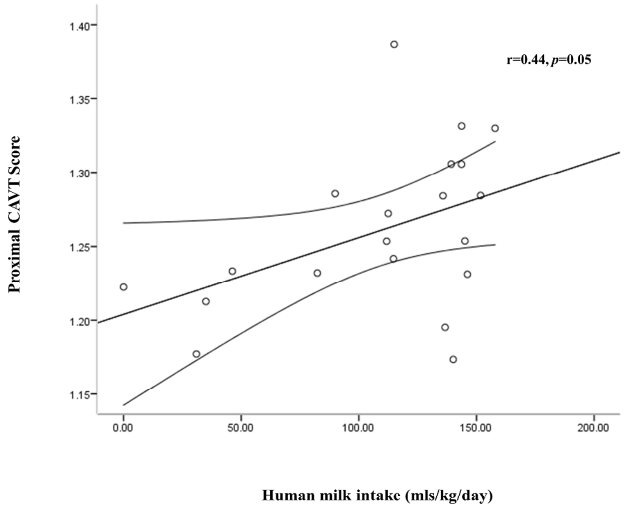


**Figure 5.** Relationship between proximal cerebral arterial vessel tortuosity (CAVT) score at term and total human milk intake (birth to 34+6 PMA) in preterm infants.

For peer review only

**Title:** Preterm nutritional intake and magnetic resonance imaging phenotype at term age: A prospective observational study.

**Authors:** Vimal Vasu[1,5] MBBS MD, Giuliana Durighel[2] MSc, Louise Thomas[2] PhD, Christina Malamateniou[3,4] PhD, Jimmy D Bell[2] PhD, Mary A Rutherford[3,4] MBBS MD, Neena Modi[1] MBChB MD

**Affiliations:** [1]Section of Neonatal Medicine, Department of Medicine, Imperial College London, Chelsea & Westminster Hospital campus, 369 Fulham Road, London, SW10 9NH, UK; [2]Metabolic and Molecular Imaging Group, MRC Clinical Sciences Centre, Imperial College London, London, W12 0HS, UK; [3]Division of Clinical Sciences, Imperial College London, MRC Clinical Sciences Centre Hammersmith Hospital, Du Cane Road, London, United Kingdom, W12 0HS, UK; [4] Centre for the Developing Brain, Department of Perinatal Imaging & Health, Division of Imaging Sciences & Biomedical Engineering, Faculty of Medicine, King's College London, St Thomas' Hospital, London SE1 7EH, UK; [5] Department of Child Health, William Harvey Hospital, Ashford, Kent, TN24 0LZ, UK.

**Address Correspondence to:** Dr Vimal Vasu, Department of Child Health, William Harvey Hospital, Ashford, Kent, TN24 0LZ, United Kingdom.

Email: vimal.vasu@nhs.net

**Short Title:** Preterm nutrition and phenotype

For peer review only

**Abbreviations:** ACA-anterior cerebral artery, AT-adipose tissue, CAVT-cerebral arterial vessel tortuosity, CRIB II-Clinical Risk Index for Babies II, CSF-Cerebrospinal fluid, DSC-deep subcutaneous, I-internal, IHCL-intrahepatocellular lipid, MCA-middle cerebral artery, MR-magnetic resonance, NMR-nuclear magnetic resonance, PMA-post menstrual age, PCA-posterior cerebral artery, SDS- standard deviation scores, SSC-superficial subcutaneous

**Key Words:** preterm, nutrition, body composition, magnetic resonance

**Funding Sources:** United Kingdom Medical Research Council; Chelsea and Westminster Hospital NHS Foundation Trust

**Financial Disclosure:** None

**Conflict of Interest:** None

**Clinical Trials Registration:** Not Applicable

**Data sharing statement:** No additional data available

**Contributorship Statement:** VV helped design the original prospective study, analysed the brain MRI data, drafted the initial manuscript and approved the final manuscript as submitted. GD optimized MR imaging parameters for the study. LT coordinated analysis of the adipose tissue MRI data. CM optimised MR angiography and advised on vessel tortuosity measurements. JB was involved in study design and

For peer review only

supervised analysis of the adipose tissue MRI data. MR was involved in study design and supervised analysis of brain MRI data. NM was involved with study design and supervised the analysis of neonatal nutritional data. All authors critically reviewed and revised the manuscript and approved the final manuscript as submitted.

For peer review only

**ABSTRACT**

**Objective** To describe (i) the relationship between nutrition and the preterm-at-term infant phenotype, (ii) phenotypic differences between preterm-at-term infants and healthy term born infants and (iii) relationships between somatic and brain MRI outcomes.

**Design** Prospective observational study.

**Setting** UK tertiary neonatal unit.

**Participants** Preterm infants (<32 weeks gestation) (n=22) and healthy term infants (n=39)

**Main outcome measures** Preterm nutrient intake; total and regional adipose tissue (AT) depot volumes; brain volume and proximal cerebral arterial vessel tortuosity (CAVT) in preterm infants and in term infants.

**Results** Preterm nutrition was deficient in protein, high in carbohydrate and high in fat. Preterm nutrition was not related to AT volumes, brain volume or proximal CAVT score; a positive association was noted between human milk intake and proximal CAVT score (r=0.44, $p$=0.05). In comparison to term infants, preterm infants had increased total adiposity, comparable brain volumes and reduced proximal CAVT scores. There was a significant negative correlation between deep subcutaneous abdominal adipose tissue volume and brain volume in preterm infants (r= -0.58, $p$=0.01).

**Conclusions** Though there are significant phenotypic differences between preterm infants at term and term infants, preterm macronutrient intake does not appear to be a determinant. Our preliminary data suggest that (i) human milk maybe exert a beneficial effect upon cerebral arterial vessel tortuosity (ii) there is a negative correlation between adiposity and brain volume in preterm infants at term. Further

For peer review only

work is warranted to see if our findings can be replicated and to understand the causal mechanisms.

For peer review only

## ARTICLE SUMMARY

- Provision of nutrition to preterm infants is complicated by immaturity, critical illness, and a limited evidence base relating early nutrition to functional health outcomes.
- In comparison to term infants, preterm infant phenotype at term is characterized by adiposity, comparable brain volume, increased CSF volume and reduced cerebral artery tortuosity.
- Though preterm macronutrient intake is not a determinant of this phenotype, human milk intake is positively associated with cerebral artery tortuosity.
- In preterm infants, regional adiposity is negatively correlated with brain volume measured at term age.

For peer review only

**Strengths of study**

- No previously published studies have studied the relationship between preterm nutritional intake and MRI outcomes at term age.
- Comprehensive ascertainment of preterm nutritional data in parallel with somatic and brain MRI.
- Use of term born infants as comparator for MRI outcomes.

**Limitations of study**

- Limited sample size.
- Prospective observational nature of study.

For peer review only

## INTRODUCTION

Preterm nutritional guidelines are based upon consensus expert opinion (1,2) rather than compelling evidence and the “optimal” diet for long term health remains unknown (3). Though there is now greater clinical emphasis upon preterm nutrition, protein deficiency remains a significant risk and our previously published data in 18 preterm infants from a single centre indicate that the preterm diet is low in protein whilst being high in both carbohydrate and fat (4). Our data also suggest that preterm macronutrition may affect later health by demonstrating a positive association between first week lipid intake in preterm infants and elevated intrahepatocellular lipid (IHCL), which in adults is associated with the cardio-metabolic syndrome (5).

The preterm phenotype at term is characterized by aberrant adipose tissue partitioning (6), reduced proximal cerebral arterial vessel tortuosity (CAVT) (7), reduced deep grey matter volumes (8), and reduced cerebral cortical folding (9). The somatic phenotype observed is of concern as adiposity is associated with inflammation and reduced brain volume in the adult population (10).

Here we present prospective observational data designed to (i) assess the influence of nutrition upon the preterm phenotype at term age (ii) describe phenotypic differences between preterm infants at term and term healthy infants and (iii) examine relationships between somatic and brain MRI measurements. The a priori hypotheses of our study were that (i) preterm macronutrient intake would be positively associated with central nervous system phenotype (brain volume & cerebral vessel tortuosity) and (ii) preterm macronutrient intake would be negatively associated with internal

For peer review only

abdominal (visceral) adiposity. The relationship between somatic and brain MRI outcomes represents a post hoc exploratory analysis.

For peer review only

## PATIENTS AND METHODS

Following research ethics approval (REC 07/Q0403/46) and informed parent consent, preterm infants admitted to Chelsea and Westminster Hospital neonatal unit, London, UK (< 32 weeks of gestation) and term infants (37-42 weeks gestation) on the postnatal ward were recruited (January 2007-July 2008). Infants with congenital anomalies were excluded.

### Preterm nutrition and growth

Preterm nutritional practice during the study period has been previously described (4). In brief, enteral feeds were commenced on day 1 with either maternal expressed breast milk or donor expressed breast milk prior to consideration of the use of formula. Human milk fed infants received Nutriprem breast milk fortifier (Cow & Gate) once 150ml/kg/day of feed volume was reached. Parenteral nutrition was commenced on the day of birth.

Weight and head circumference data for the preterm group are expressed as SDS at birth and at time of MRI. Birth length was not routinely measured during the study period. Macronutrient and human milk intake was recorded between birth and $34^{+6}$ weeks PMA using a nutritional data capture system designed in-house (Nutcracker™, Imperial College, London, UK). Macronutrient data were expressed as the difference between recommended daily intake (RDI) from Tsang et al (i) and actual daily intake in g or kcal/kg for the period of either early (first week of life) or total nutrition (birth and $34^{+6}$ PMA) (iv). Human milk intake is expressed as ml/kg/day and as percentage of enteral feeds given as human milk.

For peer review only

**Magnetic Resonance Imaging**

MRI was performed at the Robert Steiner Unit, Hammersmith Hospital shortly after discharge from the neonatal unit as previously described (iv) using a Philips Achieva system (Best, the Netherlands).

*(i) Total and Regional AT*

Imaging parameters are shown in Table 1. Regional AT depots were classified as total superficial subcutaneous, total deep subcutaneous and total internal and subdivided into abdominal or non-abdominal (Figures 1 & 2). AT volumes were calculated as previously described (6). Non AT mass was calculated by conversion of AT volume to AT mass (density of AT of 0.9 grams/$cm^3$) and subtraction of the result from weight at time of MRI.

*(ii) Brain volume*

T2 weighted images were acquired using a dynamic sequence of six separate loops of single shot images which were then registered and reconstructed to produce volumetric datasets to eliminate the effects of motion artefact (11). Imaging parameters are shown in Table 1. Images were corrected for Radiofrequency inhomogeneity (http://mipav.cit.nih.gov). BET Brain extraction tool FSL Version 3 (http://www.fmrib.ox.ac.uk/fsl/) (12) was then used to delete non brain tissue and create binary brain masks representing intracranial volume (13). A mask of ventricular and CSF spaces was created using the thresholding feature of Image J, a java based image processing program (14). Brain volume was calculated by subtraction of the volume of the ventricular and CSF mask from the volume of the intracranial mask using ImageJ (Figure 3). Brain MRIs were reported by MR for

For peer review only

clinical purposes and given a score (0-13) adapted from Dyet et al (15) so that if necessary, pathological findings could be accounted for.

*(iii) Proximal cerebral arterial vessel tortuosity (CAVT) measurement and analysis*

An optimized neonatal three dimensional time of flight MRA sequence was used to assess the anterior, middle and posterior cerebral arteries (7). Imaging parameters are shown in Table 1. Vessel tortuosity was assessed using a previously validated measurement, distance factor (16) (Figure 4) and a CAVT score was determined as a global measure of tortuosity for each subject by calculating the mean of the ACA, MCA and PCA distance factor.

**Illness severity**

CRIB II Score was calculated in preterm infants (17).

**Sample size calculation and analyses**

Based on previous work (6), we estimated that recruitment of 60 PT and 60 T infants allowed detection of a 0.5 SD difference between the groups for AT volume (Power 80%, Significance 5%). As this was an exploratory hypothesis-generating prospective observational study and given the uncertainty as to what differences were of clinical importance, additional sample size calculations were not considered. Data were adjusted for relevant confounding variables where appropriate and are presented for each of the MR outcomes with a comparison of outcomes between the groups. Data were analyzed by comparison of means and tested for normality. Parametric or non parametric methods were then applied accordingly. Within the preterm group, Pearson Correlation was used to investigate the relationship between nutrition and MR

For peer review only

outcomes at term age and these were limited to the patients in who both nutritional data and the MRI outcome were successfully acquired. For a multivariate analysis a minimum of 10n subjects is considered appropriate, where n is the number of covariates. Data are presented as mean (SD) or mean (95% CI).

For peer review only

# RESULTS

61 infants were recruited during the study (Preterm 22, Term 39). MR images without motion artefact were acquired as follows: AT volume (preterm 22, term 39), brain volume (preterm 19, term 19), CAVT measurement (preterm 20, term 13). In preterm infants, mean (SD) CRIB II score was 7.6 (4.2). 25% had chronic lung disease of prematurity (defined as an oxygen requirement at 36 weeks PMA), 20% had a patent ductus arteriosus requiring pharmacological therapy, 5% had retinopathy of prematurity requiring laser therapy and 5% had a large intraventricular haemorrhage (Grade III/IV Papile classification). 0% had necrotising enterocolitis requiring surgery. Table 2 summarises the baseline and imaging characteristics and the MR outcomes of the preterm and term groups.

## Preterm nutrition & growth

Growth and nutrition data for the preterm cohort have been previously published (4). In brief, mean (SD) for birth weight and birth head circumference SDS were: -0.13 (0.78) and -0.64 (1.12). At time of MRI, the respective values were: -1.39 (0.93) and -0.86 (1.33). Weight gain was mean (SD) 9.48 (1.73) g/kg/day between birth and $34^{+6}$ weeks. Preterm macronutrient intakes for both the first week after birth and for the period from birth until $34^{+6}$ weeks post menstrual age revealed a mean protein deficit (first week: -1.6g/kg/day; birth until $34^{+6}$ weeks -0.4g/kg/day) in the context of excessive carbohydrate and fat intake. Mean (SD) human milk intakes for these periods were 36.6 (29.7) ml/kg/day and 108.9 (46.6) ml/kg/day respectively.

For peer review only

## Preterm Nutrition and MRI Outcomes

*(i) Human milk and MRI outcomes*

After adjustment for weight at MRI, there was no correlation between early or total human milk intake and either regional AT volumes or brain volume at term age. There was no correlation between early human milk intake and overall CAVT score (r= 0.31, p=0.18); however, there was a weak positive relationship between total human milk intake and overall CAVT score (r=0.44, p=0.05) (Figure 5). This relationship was also apparent when total human milk intake was expressed as percentage of total milk intake (r=0.45, p=0.04).

*(ii) Macronutrient intake and MRI outcomes*

There were no correlations between macronutrient intake (protein, carbohydrate, fat) and total adiposity (n=22). After adjustment for PMA, neither early nor total preterm macronutrient intake were correlated with brain volume at term age (n=19). There were no relationships noted between early or total macronutrient intake and proximal CAVT score (n=20), a summary measure of tortuosity.

## Preterm at Term versus Term Group Comparison

*(i) Anthropometry*

Anthropometric data were adjusted for PMA at time of MRI. Preterm infants were smaller, shorter and had smaller head circumferences than term infants (Table 2).

For peer review only

*(ii) Adiposity*

Adipose tissue data were adjusted for PMA at time of MRI. Preterm infants had more AT than term infants with expansion of the superficial subcutaneous, deep subcutaneous and internal AT depots. There was a parallel reduction in non adipose tissue mass (Table 2).

*(iii) Brain volume*

Brain MRI scores in the cohort of preterm infants were mean (SD) 3.6 (2.1). There was no correlation between brain MRI score and brain volume (r=0.31, *p*=0.20). At term age, preterm brain volumes were smaller than term infants [PT 461.74 (436.16-487.32) ml v 493.74 (476.43-511.05) ml, *p*=0.05. However, after adjustment for weight at time of MRI, a significant confounding factor, there was no difference between the groups. Preterm-at-term infants had significantly increased CSF volume (Table 2).

*(iv) Distance Factor & Proximal CAVT score*

In comparison to term born infants, preterm-at-term infants had a significant reduction in cerebral arterial vessel tortuosity (Table 2).

**Relationships between somatic and central nervous system MRI phenotype**

After adjustment for weight at MRI, there was a significant negative correlation noted in preterm at term infants but not in term infants, between deep subcutaneous AT volume and brain volume (r=-0.58, *p*=0.01) (Table 3). There were no statistically significant correlations between regional AT and CAVT score.

For peer review only

**DISCUSSION**

We identify novel findings of interest including a positive association between human milk intake and proximal CAVT, a marker of cerebrovascular development and a negative correlation between regional adipose tissue volume and brain volume in preterm infants. The study also comprehensively characterises the somatic and brain phenotype of a cohort of preterm infants at term in comparison to term born healthy infants and demonstrates (i) reduced anthropometric measures (ii) increased total and regional adiposity (iii) reduced non-adipose tissue mass (iv) comparable brain volumes (v) increased CSF volume and (vi) reduced proximal cerebral arterial vessel tortuosity. We have shown no relationship with either body composition or brain volume at term age within the range of macronutritional intakes received by the preterm infants in this study.

Key study strengths include the comprehensive ascertainment of preterm nutritional data and the assessment of a number of MR outcomes. Limitations include the prospective observational design of the study that preclude any inferences regarding causality, and the "sub-optimal" preterm nutritional intake, a potential determinant of the observed phenotype. Successful acquisition of a number of different MR outcomes without use of sedation was challenging and motion artefact meant that not all recruited infants had data of sufficient quality for analysis. We were unable to recruit the desired number of infants and recognise that the study may be underpowered. The preterm infants studied were relatively healthy, as evidenced by low illness severity (CRIB II scores), low incidences of serious neonatal morbidity and low brain MR scores, factors that may have attenuated any associations with nutritional intake.

For peer review only

The finding of increased adiposity in preterm infants confirms our previous work (6). We have previously shown expansion of the internal abdominal AT compartment in preterm at term infants in comparison to term born infants in a cohort of preterm infants recruited in 2002-2003 (6), a time at which neonatal nutrition was possibly not as carefully considered as it is today. This contrasts with our present data which demonstrates a global expansion of all AT depots. Whether the differences observed in AT partitioning between these studies relate to changes in nutritional practises and the provision of a more calorie dense diet is a plausible but as yet unproven hypothesis. Other potential mechanisms, which we have not explored, that might explain the increased adiposity seen in preterm infants include weight cycling and inflammation. It is known that cycling between high and low calorie diets (weight cycling) (18) results in the preferential deposition of AT over non AT mass (19) and this phenomenon often occurs in preterm infants when enteral feeds are discontinued because of concerns regarding feed intolerance and then restarted. Inflammation is also a known determinant of adiposity (20) and it is possible that the pro-inflammatory milieu often present in the perinatal period (maternal chorioamnionitis, use of intravenous lipid formulations high in omega-6 fatty acids and postnatal infection/ inflammation) may be relevant to the observed phenotype.

The nutritional intake received by this cohort of preterm infants was imbalanced (low in protein and high in both fat and carbohydrate) and sub-optimal in relation to expert consensus recommendations (1,2). In animal models, protein deficiency is associated with a number of adverse health outcomes including a reduction in life span (21), cardiovascular dysfunction (22), reduced dendritic spine density (23), reduced brain

For peer review only

weight (24) and reduced cortical blood vessel density (25). Human adult data indicate that dietary protein is an important factor in body weight regulation (26). Recent data in human ex-preterm infants suggests an association between early growth patterns and fractional anisotropy, a measure of brain microstructure (27). Our group have previously shown that preterm at term AT deposition can be attenuated by use of fortified human milk (28). Whether this translates into longer term benefit is unknown.

The negative correlation we show between adiposity in preterm at term infants and brain volume is consistent with findings in adults and children (10,29,30). This notwithstanding preterm at term brain volume was comparable to that of term born healthy infants which is also in keeping with previously published work (31). This, together with the finding of maintained head circumference SDS between birth and time of imaging may be indicative of “brain sparing” in nutritionally compromised infants.

We have confirmed the finding of reduced proximal CAVT in preterm infants (7). Though the natural history and long term neurodevelopmental sequelae of reduced CAVT are unknown, epidemiological data indicate that advancing gestation confers a significant reduction in risk of fatal adult cerebrovascular disease (occlusive stroke) (32). Our observation that human milk may be protective despite low macronutrient density, suggests that non-nutritive factors, such as vascular endothelial growth factor may play an important role in cerebrovascular development.

For peer review only

In conclusion, we have extended the characterization of the preterm-at-term phenotype. Our data do not support an association between macronutrient intake and body composition or brain volume. Other plausible determinants that remain to be explored are the roles of micronutrient deficiency, weight cycling, disease severity, and chronic inflammation.

For peer review only

## FIGURE LEGENDS

**Figure 1.** Classification of adipose tissue (AT) depots into internal, superficial subcutaneous and deep subcutaneous depots and further sub-classification according to abdominal or non abdominal position.

**Figure 2.** T1 weighted axial magnetic resonance image (abdominal level) demonstrating the deep and superficial subcutaneous adipose tissue depots. A clear fascial plane is noted between the superficial and deep subcutaneous layers (arrows).

**Figure 3.** a. T2 weighted MR image undergoes RF inhomogeneity correction and subsequent creation of binary brain mask of intracranial volume (BET brain extraction tool FSL version 3). b. Creation of a mask of the ventricular and cerebrospinal fluid spaces using the thresholding feature of ImageJ version 1.38. c. Calculation of actual brain volume by subtraction of ventricular & CSF volume from intracranial volume.

**Figure 4.** a. Axial magnetic resonance angiogram demonstrating tracing of a contour along the left middle cerebral artery for 30mm (black line) along with a straight line measurement between the start point and the end point of the traced contour (red line). b. Sagittal magnetic resonance angiogram demonstrating tracing of a contour along the anterior cerebral artery for 30mm (black line) along with a straight line measurement between the start point and the end point of the traced contour (red line).

For peer review only

**Figure 5.** Relationship between proximal cerebral arterial vessel tortuosity (CAVT) score at term and total human milk intake (birth to $34^{+6}$ weeks PMA) in preterm infants.

For peer review only

**Table 1.** MR imaging parameters for adipose tissue, brain volume and proximal cerebral arterial vessel tortuosity.

| | Planes | Weighting | FOV | Slices | Slice thickness | Slice gap | TE | TR | Acquisition time |
|---|---|---|---|---|---|---|---|---|---|
| **Adipose tissue**[6] | Axial | T1 Fast Spin Echo | 300 | 25 | 5mm | 5mm | 20ms | 500ms | 360s |
| **Brain volume**[11] | Axial/Sagittal | T2 | 220 | 60 | 3mm | -1.5mm | 160ms | 16646/38662ms | 425s |
| **Proximal cerebral arterial vessel tortuosity**[7] | Axial/Sagittal | Angiogram | 175 | 100 | 0.6mm | 0mm | 8.06ms | 19ms | 329s |

For peer review only

**Table 2.** Birth characteristics, imaging characteristics and MRI outcomes in preterm-at-term and term infants.

| | Preterm-at-Term | n | Term | n | 95% CI for Difference |
|---|---|---|---|---|---|
| **Birth Characteristics** | | | | | |
| Gestation at Birth (weeks) | 28.9 (2.8) | 22 | 40.2 (1.20) | 39 | N/A |
| Birth Weight (kg) | 1.26 (0.41) | 22 | 3.40 (0.45) | 39 | N/A |
| Gestation at MRI (weeks) | 40.2 (3.10) | 22 | 41.4 (1.40) | 39 | N/A |
| | | | | | |
| **Anthropometry at time of MRI** | | | | | |
| Weight at MRI (kg) | 3.01 (2.83-3.19) | 22 | 3.30 (3.17-3.44) | 39 | 0.30 (0.66-0.53)* |
| Length at MRI (cm) | 47.9 (46.9-48.9) | 22 | 51.3 (50.5-52.1) | 39 | 3.4 (2.1-4.7)** |
| Head Circumference at MRI (cm) | 34.2 (33.6-34.7) | 22 | 35.2 (34.8-35.6) | 39 | 1.1 (0.4-1.7)** |
| | | | | | |
| **Somatic MRI Outcomes** | | | | | |
| Total AT (l) | 0.782 (0.735-0.829) | 22 | 0.657 (0.623-692) | 39 | 0.125 (0.064-0.186)** |
| Total Deep Subcutaneous AT (l) | 0.033 (0.029-0.036) | 22 | 0.024 (0.021-0.027) | 39 | 0.009 (0.004-0.013)** |
| Total Superficial Subcutaneous AT (l) | 0.673 (0.631-0.715) | 22 | 0.568 (0.538-0.599) | 39 | 0.105 (0.051-0.159)** |
| Total Internal AT (l) | 0.076 (0.069-0.083) | 22 | 0.065 (0.060-0.070) | 39 | 0.011 (0.002-0.020)* |
| Deep Subcutaneous Adipose Abdominal AT (l) | 0.017 (0.014-0.019) | 22 | 0.012 (0.011-0.014) | 39 | 0.004 (0.001-0.005)** |
| Deep Subcutaneous Adipose Non Abdominal AT (l) | 0.016 (0.014-0.018) | 22 | 0.012 (0.010-0.013) | 39 | 0.004 (0.002-0.007)** |
| Superficial Subcutaneous Adipose Abdominal AT (l) | 0.133 (0.122-0.145) | 22 | 0.091 (0.083-0.100) | 39 | 0.042 (0.027-0.057)** |
| Superficial Subcutaneous Adipose Non Abdominal AT (l) | 0.540 (0.506-0.574) | 22 | 0.477 (0.452-0.502) | 39 | 0.063 (0.019-0.107)** |
| Internal Abdominal AT (l) | 0.022 (0.019-0.025) | 22 | 0.018 (0.016-0.021) | 39 | 0.004 (0-0.007) |
| Internal Non Abdominal AT (1) | 0.054 (0.049-0.060) | 22 | 0.047 (0.043-0.051) | 39 | 0.008 (0.001-0.005)* |
| Non AT mass (kg) | 2.49 (2.45-2.54) | 22 | 2.61 (2.57-2.64) | 39 | 0.12 (0.06-0.170)** |
| | | | | | |
| **Brain MRI Outcomes** | | | | | |
| Brain Volume (ml) | 481.46 (462.29-498.63) | 19 | 474.02 (456.85-491.20) | 19 | 7.44 (18.53-33.40) |
| Cerebrospinal Fluid Volume (ml) | 63.78 (55.06-72.50) | 19 | 31.44 (22.72-40.16) | 19 | 32.34 (19.15-45.53)** |
| Anterior Cerebral Artery DF | 1.38 (1.30-1.46) | 20 | 1.25 (1.19-1.32) | 13 | 0.13 (0.03-0.23)* |
| Middle Cerebral Artery DF | 1.38 (1.32-1.44) | 20 | 1.26 (1.21-1.31) | 13 | 0.12 (0.04-0.19)** |
| Posterior Cerebral Artery DF | 1.46 (1.41-1.50) | 20 | 1.27 (1.24-1.30) | 13 | 0.19 (0.14-0.24)** |
| Proximal CAVT score | 1.41 (1.36-1.45) | 20 | 1.26 (1.23-1.29) | 13 | 0.14 (0.09-0.20)** |

*Footnotes: Results are mean (SD) or mean (95% CI). * p<0.05, **p<0.01. (AT-adipose tissue, DF- distance factor, CAVT- cerebral arterial vessel tortuosity, n- sample size).*

For peer review only

**Table 3.** Pearson correlations between regional adipose tissue (AT) depots and brain volume in preterm at term and term infants adjusted for weight at imaging.

| **Regional AT depot** | **Preterm at Term (n=19)** | **Term (n=19)** |
|---|---|---|
| Total AT | -0.23, p=0.38 | -0.23, p=0.35 |
| Total DSC AT | -0.58, p=0.01* | -0.05, p=0.85 |
| Total SSC AT | -0.22, p=0.39 | -0.24, p=0.34 |
| Total I AT | -0.09, p=0.73 | -0.13, p=0.96 |

*AT: Total adipose tissue, Total I AT:Total internal adipose tissue, Total SSC AT:Total superficial subcutaneous adipose tissue, Total DSC AT:Total deep subcutaneous adipose tissue, IA AT:Internal abdominal adipose tissue.*

For peer review only

## REFERENCES


1. Tsang R, Uauy R, Koletzko B et al. Nutrition of the preterm infant: Scientific basis and practical guidelines. 2nd ed. Cincinnati, OH: Digital Education Publishing, Inc; 2005: 415-416.

2. Agostoni C, Buonocore G, Carnielli VP et al. Enteral nutrient supply for preterm infants: commentary from the European Society of Paediatric Gastroenterology, Hepatology and Nutrition Committee on Nutrition. *J Pediatr Gastroenterol Nutr*. 2010;50(1):85-91.

3. Vasu V, Modi N. Assessing the impact of preterm nutrition. *Early Hum Dev*. 2007;83(12):813-818.

4. Vasu V, Thomas EL, Durighel G et al. Early Nutritional Determinants of Intrahepatocellular Lipid Deposition in Preterm Infants at Term Age. *Int J Obes (Lond)*. 2013;37(4);500-504.

5. Mehta SR, Godsland IF, Thomas EL et al. Intrahepatic insulin exposure, intrahepatocellular lipid and regional body fat in nonalcoholic fatty liver disease. *J Clin Endocrinol Metab*. 2012;97(6):2151-2159.

6. Uthaya S, Thomas EL, Hamilton G et al. Altered adiposity after extremely preterm birth. *Pediatr Res*. 2005;57(2):221-225.


For peer review only

7. Malamateniou C, Counsell SJ, Allsop JM et al. The effect of preterm birth on neonatal cerebral vasculature studied with magnetic resonance angiography at 3 Tesla. *Neuroimage*. 2006;32(3):1050-1059.

8. Srinivasan L, Dutta R, Counsell SJ et al. Quantification of deep gray matter in preterm infants at term-equivalent age using manual volumetry of 3-tesla magnetic resonance images. *Pediatrics*. 2007;119:759-765.

9. Ajayi-Obe M, Saeed N, Cowan FM et al. Reduced development of cerebral cortex in extremely preterm infants. *Lancet*. 2000;356(9236):1162-1163.

10. Debette S, Wolf C, Lambert JC et al. Abdominal obesity and lower gray matter volume: a Mendelian randomization study. Neurobiology of Aging. 2014;35(2):378-386. Epub 2013 Aug 31.

11. Jiang S, Xue H, Glover A et al. MRI of moving subjects using multislice snapshot images with volume reconstruction (SVR): application to fetal, neonatal, and adult brain studies. *IEEE Trans Med Imaging*. 2007;26(7):967-980.

12. Smith SM, Jenkinson M, Woolrich MW et al. Advances in functional and structural MR image analysis and implementation as FSL. *Neuroimage*. 2004;23 Suppl 1:S208-219.

13. Smith SM. Fast robust automated brain extraction. *Hum Brain Mapp*. 2002;17(3):143-155.

For peer review only

14. Rasband, W.S. ImageJ, U. S. National Institutes of Health, Bethesda, Maryland, USA, http://imagej.nih.gov/ij/, 1997-2012.

15. Dyet LE, Kennea N, Counsell SJ et al. Natural History of Brain Lesions in Extremely Preterm Infants Studied With Serial Magnetic Resonance Imaging From Birth and Neurodevelopmental Assessment. *Pediatrics*. 2006;118:536 - 548.

16. Bullitt E, Gerig G, Pizer SM et al. Measuring tortuosity of the intracerebral vasculature from MRA images. *IEEE Trans Med Imaging*. 2003;22(9):1163-1171.

17. Parry G, Tucker J, Tarnow-Mordi W. CRIB II : an update of the Clinical Risk Index for Babies score. *Lancet*. 2003;361:1789-1791.

18. Brownell KD, Greenwood MR, Stellar E et al. The effects of repeated cycles of weight loss and regain in rats. *Physiol Behav*. 1986;38(4):459-464.

19. Rodin J, Radke-Sharpe N, Rebuffé-Scrive M et al. Weight cycling and fat distribution. *Int J Obes*. 1990;14:303-310.

20. So PW, Herlihy AH, Bell JD. Adiposity induced by adenovirus inoculation. *Int J Obes (Lond)*. 2005;29(6):603-606.

For peer review only

21. Ozanne SE, Hales CN. Lifespan: Catch-up growth and obesity in male mice. *Nature*. 2004;427(6973):411-412.

22. Cleal JK, Poore KR, Boullin JP et al. Mismatched pre- and postnatal nutrition leads to cardiovascular dysfunction and altered renal function in adulthood. *PNAS*. 2007;104(22):9529-9533.

23. Garcia-Ruiz M, az-Cintra S, Cintra L et al. Effect of protein malnutrition on CA3 hippocampal pyramidal cells in rats of three ages. *Brain Res*. 1993;625(2):203-12.

24. Perez-Torrero E, Torrero C, Salas M. Effects of perinatal undernourishment on neuronal development of the facial motor nucleus in the rat. *Brain Res*. 2001;905:54-62.

25. Bennis-Taleb N, Remacle C, Hoet JJ et al. A Low-Protein Diet During Gestation Affects Brain Development and Alters Permanently Cerebral Cortex Blood Vessels in Rat Offspring. *J Nutr*. 1999;129(8):1613-1619.

26. Westerterp-Plantenga MS, Lejeune MP, Nijs I et al. High protein intake sustains weight maintenance after body weight loss in humans. *Int J Obes Relat Metab Disord*. 2004:28(1):57–64.

27. Vinall J, Grunau RE, Brant R et al. Slower postnatal growth is associated with delayed cerebral cortical maturation in preterm newborns. *Sci Transl Med*. 2013;5(168):168ra8.

For peer review only

28. Uthaya S, Thomas EL, Bell J et al. Improved growth and adipose tissue partitioning in preterm infants receiving exclusive enteral feeds of fortified human milk in comparison with infants receiving human milk and formula.Proceedings of the Neonatal Society. 2007. (http://www.neonatalsociety.ac.uk/abstracts/uthayas_2007_growthfortifiedfeeds.shtml - 7KB)

29. Yau PL, Castro MG, Tagani A et al. Obesity and metabolic syndrome and functional and structural brain impairements in adolescence. Pediatrics. 2012;130(4):e856-864.

30. Debette S, Beiser A, Hoffmann U et al. Visceral fat is associated with lower brain volume in healthy middle-aged adults. *Ann Neurol*. 2010;68(2):136-144.

31. Boardman JP, Counsell SJ, Rueckert D et al. Early growth in brain volume is preserved in the majority of preterm infants. *Ann Neurol*. 2007;62(2):185-192

32. Koupil I, Leon DA, Lithell HO. Length of gestation is associated with mortality from cerebrovascular disease. *J Epidemiol Community Health*. 2005;59:473-474.

For peer review only

STROBE Statement—Checklist of items that should be included in reports of ***cohort studies***

| | Item No | Recommendation |
|---|---|---|
| **Title and abstract** | 1 | (*a*) Indicate the study's design with a commonly used term in the title or the abstract |
| | | (*b*) Provide in the abstract an informative and balanced summary of what was done and what was found |
| **Introduction** | | |
| Background/rationale | 2 | Explain the scientific background and rationale for the investigation being reported |
| Objectives | 3 | State specific objectives, including any prespecified hypotheses |
| **Methods** | | |
| Study design | 4 | Present key elements of study design early in the paper |
| Setting | 5 | Describe the setting, locations, and relevant dates, including periods of recruitment, exposure, follow-up, and data collection |
| Participants | 6 | (*a*) Give the eligibility criteria, and the sources and methods of selection of participants. Describe methods of follow-up |
| | | (*b*) For matched studies, give matching criteria and number of exposed and unexposed |
| Variables | 7 | Clearly define all outcomes, exposures, predictors, potential confounders, and effect modifiers. Give diagnostic criteria, if applicable |
| Data sources/ measurement | 8* | For each variable of interest, give sources of data and details of methods of assessment (measurement). Describe comparability of assessment methods if there is more than one group |
| Bias | 9 | Describe any efforts to address potential sources of bias |
| Study size | 10 | Explain how the study size was arrived at |
| Quantitative variables | 11 | Explain how quantitative variables were handled in the analyses. If applicable, describe which groupings were chosen and why |
| Statistical methods | 12 | (*a*) Describe all statistical methods, including those used to control for confounding |
| | | (*b*) Describe any methods used to examine subgroups and interactions |
| | | (*c*) Explain how missing data were addressed |
| | | (*d*) If applicable, explain how loss to follow-up was addressed |
| | | (*e*) Describe any sensitivity analyses |
| **Results** | | |
| Participants | 13* | (a) Report numbers of individuals at each stage of study—eg numbers potentially eligible, examined for eligibility, confirmed eligible, included in the study, completing follow-up, and analysed |
| | | (b) Give reasons for non-participation at each stage |
| | | (c) Consider use of a flow diagram |
| Descriptive data | 14* | (a) Give characteristics of study participants (eg demographic, clinical, social) and information on exposures and potential confounders |
| | | (b) Indicate number of participants with missing data for each variable of interest |
| | | (c) Summarise follow-up time (eg, average and total amount) |
| Outcome data | 15* | Report numbers of outcome events or summary measures over time |
| Main results | 16 | (*a*) Give unadjusted estimates and, if applicable, confounder-adjusted estimates and their precision (eg, 95% confidence interval). Make clear which confounders were adjusted for and why they were included |
| | | (*b*) Report category boundaries when continuous variables were categorized |
| | | (*c*) If relevant, consider translating estimates of relative risk into absolute risk for a meaningful time period |

For peer review only

| | | |
|---|---|---|
| Other analyses | 17 | Report other analyses done—eg analyses of subgroups and interactions, and sensitivity analyses |
| **Discussion** | | |
| Key results | 18 | Summarise key results with reference to study objectives |
| Limitations | 19 | Discuss limitations of the study, taking into account sources of potential bias or imprecision. Discuss both direction and magnitude of any potential bias |
| Interpretation | 20 | Give a cautious overall interpretation of results considering objectives, limitations, multiplicity of analyses, results from similar studies, and other relevant evidence |
| Generalisability | 21 | Discuss the generalisability (external validity) of the study results |
| **Other information** | | |
| Funding | 22 | Give the source of funding and the role of the funders for the present study and, if applicable, for the original study on which the present article is based |

*Give information separately for exposed and unexposed groups.

**Note:** An Explanation and Elaboration article discusses each checklist item and gives methodological background and published examples of transparent reporting. The STROBE checklist is best used in conjunction with this article (freely available on the Web sites of PLoS Medicine at http://www.plosmedicine.org/, Annals of Internal Medicine at http://www.annals.org/, and Epidemiology at http://www.epidem.com/). Information on the STROBE Initiative is available at http://www.strobe-statement.org.

For peer review only
